# Application of Halogen Bonding to Organocatalysis: A Theoretical Perspective

**DOI:** 10.3390/molecules25051045

**Published:** 2020-02-26

**Authors:** Hui Yang, Ming Wah Wong

**Affiliations:** Department of Chemistry, National University of Singapore, 3 Science Drive 3, Singapore 117543, Singapore; chmyang@nus.edu.sg

**Keywords:** halogen bond, noncovalent interaction, organocatalysis, density functional theory (DFT), mechanism, supramolecular chemistry

## Abstract

The strong, specific, and directional halogen bond (XB) is an ideal supramolecular synthon in crystal engineering, as well as rational catalyst and drug design. These attributes attracted strong growing interest in halogen bonding in the past decade and led to a wide range of applications in materials, biological, and catalysis applications. Recently, various research groups exploited the XB mode of activation in designing halogen-based Lewis acids in effecting organic transformation, and there is continual growth in this promising area. In addition to the rapid advancements in methodology development, computational investigations are well suited for mechanistic understanding, rational XB catalyst design, and the study of intermediates that are unstable when observed experimentally. In this review, we highlight recent computational studies of XB organocatalytic reactions, which provide valuable insights into the XB mode of activation, competing reaction pathways, effects of solvent and counterions, and design of novel XB catalysts.

## 1. Introduction

**Figure 1:**
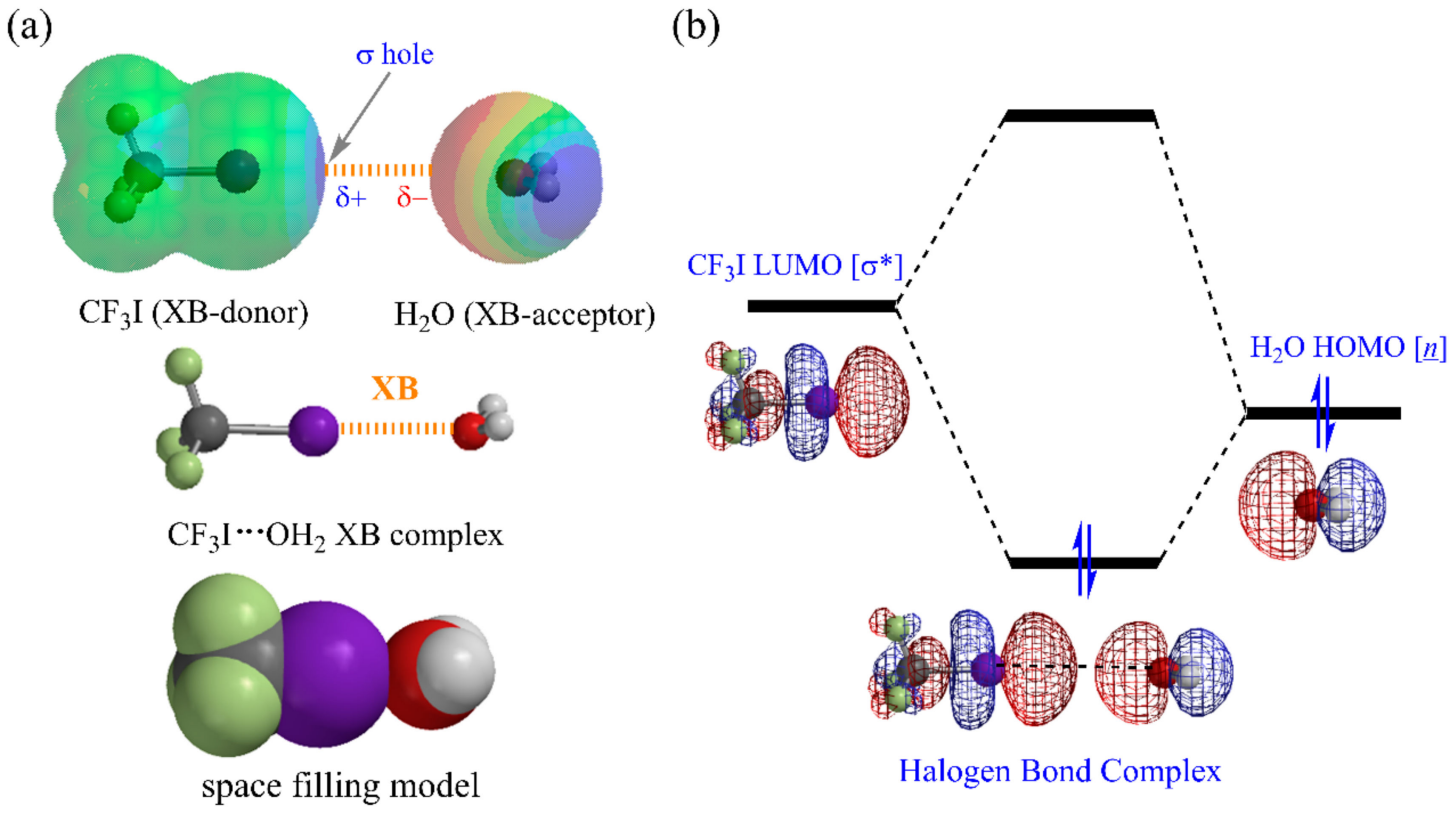
(**a**) Electrostatic (σ-hole) and (**b**) molecular orbital models of halogen bond (XB) formation. The formation of an XB complex between CF_3_I (XB-donor) and H_2_O (XB-acceptor) is used as an illustration.

Halogen bonding attracted growing interest in recent years, due to its wide range of applications as highly directional motifs in supramolecular chemistry, crystal engineering, materials science, organocatalysis, and drug design [1]. Halogen bond (XB) is defined by the International Union of Pure and Applied Chemistry (IUPAC) as “a net attractive interaction between an electrophilic region associated with a halogen atom in a molecular entity and a nucleophilic region in another, or the same, molecular entity” [2]. Essentially, the halogen atom serves as an electron-deficient moiety (XB-donor) to interact with a nucleophilic region of a Lewis base (XB-acceptor) to form a halogen bond (Figure 1). Since XB interaction features high directionality, it has excellent molecular recognition ability.

The σ-hole concept of Politzer et al. [3,4] represents the most widely accepted model to explain the origin of the halogen bond. In this model, the halogen atom is characterized by anisotropic distribution of electron density with a localized region of positive electrostatic potential (ESP) along the extension of the R–X bond (Figure 1). This model readily accounts for the close contact between halogen and nucleophile (Nu), as well as the linearity of the R–X^…^Nu angle. The magnitude of the σ-hole of XB-donors, which affects the strength of XB, depends on two key electronic factors: (i) polarizability of the halogen atom (X = I > Br > Cl > F), and (ii) electron-withdrawing ability of the group bonded to the halogen. The origin of halogen bond can also be explained by molecular orbital theory [5,6,7]. The σ* orbital of a halogen atom (i.e., the lowest unoccupied molecular orbital (LUMO) of R–X) interacts with an electron pair (lone pair or π bond) of a nucleophile (i.e., the highest occupied molecular orbital (HOMO) of Nu) leading to stabilizing the HOMO–LUMO interaction (Figure 1). The stronger frontier orbital interaction derives from the lower energy of the σ* LUMO of R–X. Thus, this model also explains the trend of XB strength for XB-donors I > Br > Cl > F and linearity of XB interaction. It is important to note that the molecular orbital interaction provides a proper description of the charge transfer contribution in XB formation. Various energy decomposition analyses showed strong supporting evidence for the role of charge transfer [8,9,10]. In particular, the frontier orbital interaction explains the site specificity of XB involving aromatic XB-acceptors, while the electrostatic model based on ESP fails to explain the site specificity [5]. Intriguingly, a few examples of strong halogen bonds with a large degree of covalency were reported [11,12,13], e.g., the N∙∙∙Br bond in bis(3-methylpyridine)bromonium perchlorate [13]. The XB interaction of this type is better represented as a coordinate (dative covalent) bond (XBA→XBD), rather than the significantly less covalent interaction (XBA∙∙∙XBD). We note that all these strong XB-bonded systems are charge-assisted XB interactions. The molecular orbital model (Figure 1) readily explains the strong donor–acceptor interaction with a large degree of charge transfer.

The parallel between the hydrogen bond (HB) and halogen bond in terms of high directionality and strength is well recognized [7,14,15]. Although the XB is generally weaker than the corresponding HB, XB has a stronger directionality. In addition, XB has a unique feature of tenability of the halogen donor as compared to hydrogen in HB. As a consequence, the halogen bonding interaction is considered to be a promising alternative to the hydrogen bonding interaction in catalysis. The haloperfluoroalkane-catalyzed reduction of quinoline derivatives using Hantzsch ester by Bolm et al. in 2008 represents the seminal work in applying XB to organocatalysis [16]. Subsequently, several groups made substantial contributions to halogen bonding in organocatalysis. These authors developed a variety of XB-donors bearing strong electron-withdrawing moieties such as perfluoro-aryls or cationic heterocycles to form strong XB with Lewis basic substrates. This led to the development of new methods in organic synthesis, e.g., nucleophilic substitution/addition, cyclization, and transfer hydrogenation reactions. Several recent excellent reviews were published on the experimental studies on XB organocatalysis [17,18]. As this review focuses on the computational studies on XB organocatalysis, we do not discuss the experimental studies in detail and ask the readers to read these reviews to appreciate the progress in recent years.

**Scheme 1:**
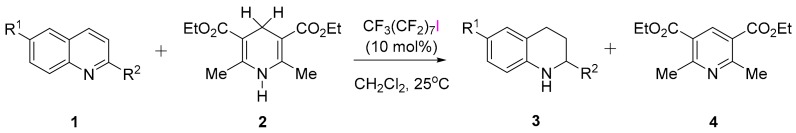
Perfluoroidoalkane (**5**)-catalyzed reduction of quinolines (**1**) by Hantzsch ester (**2**).

In the early proof-of-principle stage of halogen bond catalysis, starting from the pioneering work of Bolm et al. (Scheme 1) [16], mechanistic studies were almost exclusively conducted in experiments. The catalysts’ ability to form halogen bonds was unambiguously established via X-ray crystallography studies [19,20,21,22,23,24,25,26,27,28,29], which clearly showed the close contacts between halogen atoms and counter anions in the case of ionic catalysts, or other XB-acceptors in the case of neutral ones. In addition, NMR studies allowed probing the interaction between XB catalysts and substrates in various organic solvents [30,31]. Together with isothermal calorimetric titration [21,32], these experiments allowed the binding constants of XB-donors and XB-acceptors to be experimentally determined. Some of the largest binding constants determined experimentally are on the order of 10^3^ M^−1^ at room temperature, e.g., XB binding between tridentate polyfluorinated XB-donors and nitrogen-based XB-acceptors in non-polar organic solvents [22]. Taken together, these experimental studies provided strong support to the hypothesized halogen bond binding, which is the basis of XB catalysis. Furthermore, carefully designed control experiments were conducted, whose results showed that the halogen bond catalysis pathway was probably the most favorable one among a few other viable competitive pathways, e.g., Brønsted acid pathway and radical pathway [19,33,34].

Computational chemistry provides a useful and complementary role in understanding the mechanistic features of XB catalysis. Firstly, reliable quantum chemical calculations aid in understanding the thermodynamics and kinetics of catalytic reactions. Secondly, theory enables the characterization of the transition state, which is crucial in the understanding of the XB mode of activation and stereoselectivity. Thirdly, electronic structure calculations help to understand the governing factors in stabilizing the transition state, which in turn shed light on the origin of reactivity and stereoselectivity. Finally, theoretical calculations can be employed to design novel XB catalysts. Numerous computational studies on XB-based organocatalytic reactions demonstrated that quantum chemical calculations provide important insight into the XB mode of activation, competing reaction pathways, effects of solvent and counterions, and design of novel XB catalysts. Herein, we present a brief review of recent computational studies on this subject. Special emphasis is placed on theoretical calculations which elucidate the role of XB activation, allowing for a careful comparison with uncatalyzed reactions and competing reaction pathways.

## 2. Computational Methods for XB Organocatalysis

Over the past decade, a large number of computational studies were reported for halogen-bonded systems and various XB applications. One of the most accurate quantum chemical methods is CCSD(T), coupled cluster with single, double, and perturbative triple excitations [35]. Unfortunately, it is expensive and not practical for normal application studies. Thus, it is mainly used for benchmarking purposes for small halogen-bonded systems [36,37,38,39]. Density functional theory (DFT) methods are popular alternatives because they provide sufficient accuracy with significantly lower computational expense. Based on an extensive comparison of wave function and DFT methods on geometries and dissociation energies of halogen-bonded systems, Kozuch and Martin concluded that functionals with high exact exchange or long-range corrections, e.g., M06-2X [40], ωB97XD [41], and double hybrids, are suitable [36]. As the M06-2X functional and other latest versions of Minnesota functionals, e.g., MN15 [42], are well parametrized for noncovalent interactions and kinetics [36,37,38], M06-2X is a popular choice for studying XB organocatalysis [43]. For liquid-phase studies, the SMD universal solvation model [44] is a popular method to study the implicit solvation effect. Engelage et al. showed that the standard radii for bromine and iodine are incorrect, and the authors redefined the SMD parameters for these two halogen atoms [45]. While the majority of the reviewed papers in this article employed DFT methods, we note that the recently developed domain-based local pair–natural coupled cluster (DLPNO-CCSD(T)) method [46] is a very promising method in terms of speed and accuracy [47]. The DLPNO-CCSD(T) energy reproduces more than 99.9% of the CCSD(T) correlation energy at a computational cost of only 2–4 times that of a typical DFT method [48]. Bickelhaupt and co-workers applied this state-of-the-art method in a recent computational study on halogen bond organocatalysis [49].

To gauge the performance of various computational methods, we computed here the XB binding free energies (ΔG_298_) between iodopentafluorobenzene and triethylamine using a few popular DFT and Grimme’s dispersion-corrected [50] functionals, namely, B3LYP, B3LYP-D3, M06-2X, MN15, ω-B97XD, PBE0, and PBE0-D3, together with a moderately large 6-311++G(2d,p) basis set (Table 1). These values were benchmarked against an experimentally determined binding free energy at 298 K in cyclohexane [51]. As evidenced in Table 1, most DFT-calculated binding free energies (ΔG_298_ in cyclohexane) are in agreement with the experimental value except for the methods without dispersion correction. Recent theoretical studies [38] highlighted the importance of dispersion for accurate description of halogen bonding interactions. It is important to note that the entropic corrections to binding free energy are fairly large (9–10 kcal/mol) for all DFT methods (Table 1). Thus, it is essential to include the entropic contribution for comparison with experiment.

## 3. Computational XB Organocatalysis Studies

### 3.1. Transition State and Binding XB Complex Studies

**Figure 2:**
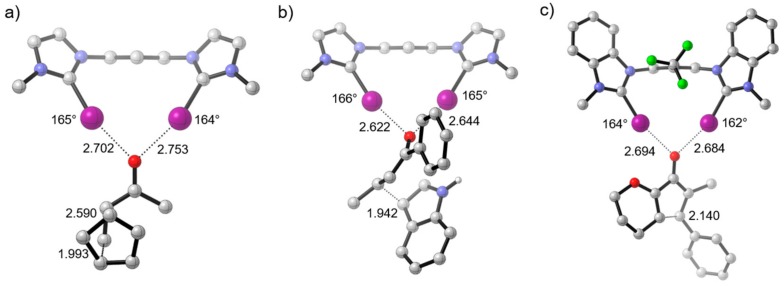
Calculated transition states for halogen bond-catalyzed (**a**) Diels–Alder cycloaddition [52], (**b**) Michael addition of indole to *trans*-crotonophenone [53], and (**c**) Nazarov cyclization reaction [54]. XB distances are in Å and A–I···B angles are in degrees. Hydrogen atoms are omitted for clarity.

Along with the experimental development of halogen bond catalysis, computational studies are playing an increasingly important role in establishing XB catalysis. For many catalyzed reactions reported by Huber et al., calculations of catalyzed transition states (TSs) or binding complexes were often performed to further support the hypothesized XB catalysis pathway (Figure 2) [23,52,53,54,55,56]. For the investigated halogen bond catalysts, especially those of bidentate and tridentate nature, calculated structures helped chemists to gain important insights, e.g., the number of halogen bonds formed and XB-acceptor atoms involved, XB interaction distances, and structural changes upon binding. These important structural insights are difficult to obtain through experimental means. While computational studies calculating XB-catalyzed TSs and/or binding complexes will continue to be an important tool to help understand XB activation, we believe that there is high demand for fuller and more comprehensive computational studies of reaction mechanisms, particularly those that calculate and compare different possible pathways. Insights from such studies could help understand the anion effect, catalyst substituent effect, solvent effect, and reaction kinetics. When combined with experiments, they can potentially lead to discovery of better catalysts. Currently, there are only a small number of such computational studies. Not surprisingly, the iodine atom, being the strongest XB-donor among the halogens, is the focus of almost all the studies.

At the start of halogen bond catalysis, the catalysts employed were neutral XB-donors [16,20,57]. At length, it was discovered experimentally that charge-enhanced XB catalysts are more potent donors than the charge-neutral ones, as the latter often failed to give any detectable product yields in a few reported experimental attempts [19,21,23,25,58]. Despite the comparatively weaker donor strength, neutral XB catalysts have several advantages over cationic catalysts. They dissolve better in organic solvents, and they are more stable and less prone to decomposition or other side reactions. Most importantly, there is less mechanistic consideration regarding the mode of activation of a neutral XB catalyst than a cationic type.

Among the neutral XB catalysts, molecular iodine, which is perhaps the simplest iodine-based donor, was an active reagent in a wide range of organic reactions [59,60,61,62,63,64]. The earliest example of catalysis by molecular iodine in the dehydration of diacetone alcohol was discovered back in 1915 [65]. Other catalytic reactions followed. However, the mechanisms of these catalytic reactions were often not well understood, and different mechanisms were often proposed for the same reactions, all with partial experimental support. While the majority of halogen bonds reported so far are based on iodine (I), there are a few studies of hypervalent iodine(III)-catalyzed reactions [57,66]. The activation of substrates was also proposed to originate from the halogen bond.

In this review, we cover and discuss iodine-based catalysts in three sections, emphasizing the mechanistic aspects, especially in terms of a comparison to uncatalyzed and other plausible side reactions. The first section deals with computational mechanistic studies of neutral halogen bond catalysts, including molecular iodine, the second section deals with ionic XB catalysts, and the third section deals with hypervalent iodine(III) catalysts.

### 3.2. Neutral Halogen Bond Catalysts

#### 3.2.1. Computational Study of XB-Catalyzed Hydrocyanation of Imines

**Scheme 2:**
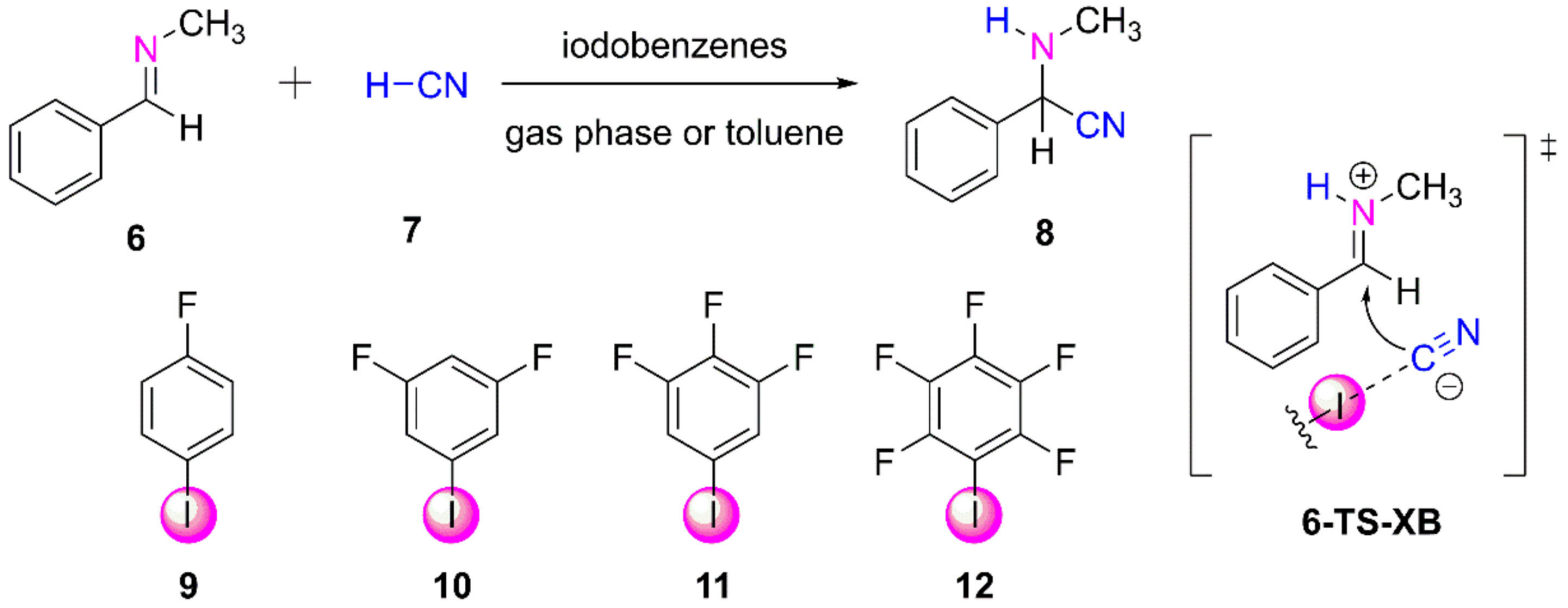
Fluoroiodobenzene-catalyzed hydrocyanation of imines.

Hydrocyanation of imines is an important reaction to synthesize amino acids [67]. Recently, Heinz et al. reported a computational investigation of feasibility of the reaction being catalyzed by neutral fluoroiodobenzene catalysts **9**–**12** (Scheme 2) [68].

The DFT method BP86 [69], together with the def2-TZVP basis set for all atoms and ecp-28-mdf pseudopotential for iodine, was used. Both gas- and solution-phase energies were computed. In the latter case, the conductor-like screening model (COSMO) polarizable continuum model (PCM) was employed to study the solvent effect of the calculated gas-phase structures, with toluene used as a solvent. Zero-point energy correction was included in the calculated energies.

**Figure 3:**
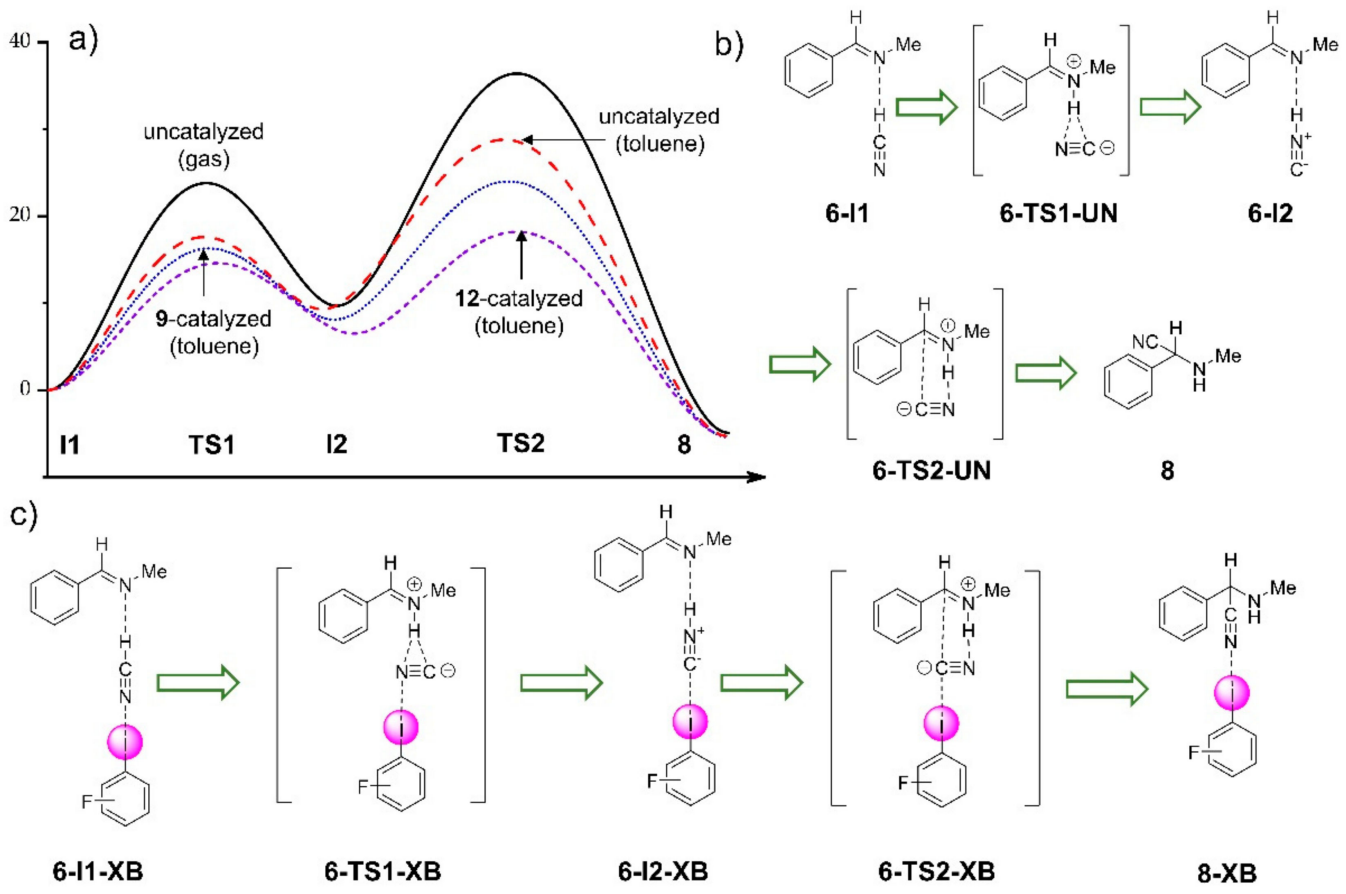
Schematic reaction profiles of (**a**) uncatalyzed hydrocyanation of imine in the gas phase (black solid line), in toluene (red dashed line), **9**-catalyzed in toluene (blue dotted line), and **12**-catalyzed in toluene (purple short dashed line); (**b**) mechanism of the uncatalyzed reaction; (**c**) mechanism of the XB-catalyzed reaction.

The authors firstly investigated the uncatalyzed hydrocyanation reaction in both the gas phase and in toluene (Figure 3). Catalyzed reactions by four XB catalysts (**9**–**12**) were next investigated, and results indicated that all catalysts lowered the activation energy in toluene significantly, with **12**, having the most electron-withdrawing aryl ring, lowering it the most by 10.6 kcal/mol and **9** the least by 4.8 kcal/mol.

The reduction of reaction barrier of hydrocyanation reaction of imine by 4–10 kcal/mol via XB catalysis using various fluoroiodobenzenes (**9**–**12**) seems to be a remarkable result, given their monodentate nature. Experimentally, only in a very few cases were monodentate XB compounds found to be catalytic [16,26,70]. However, these calculations were performed without considering the effect of entropy (see Table 1), which cannot be ignored when comparing catalyzed to uncatalyzed reactions. Furthermore, the reference points of the reaction profiles were chosen to be the weakly hydrogen bonded (**6-I1**) or halogen-bonded complexes (**6-I1-XB**) rather than the usual free reactants and catalysts. Hence, the conclusion is likely to change upon inclusion of entropy correction and optimization in a solvent medium.

#### 3.2.2. Computational Design of Neutral XB Catalysts

In 2016, our group reported a computational study of an in silico designed tridentate halogen bond catalyst, with due consideration of reaction free energies and reaction kinetics [71]. All the calculations were performed using the M06-2X functional. For geometry optimizations, a small basis set, 6-31G(d) for non-iodine atoms and aug-cc-pVTZ for iodine, referred to as the SMALL basis set, was used, while, for single-point energy calculations, a large one, 6-311+G(d,p) for non-iodine atoms and aug-cc-pVTZ for iodine, referred to as the BIG basis set, was used. Reported relative free energies correspond to the M06-2X/BIG//M06-2X/SMALL level of theory at 298.15 K.

**Figure 4:**
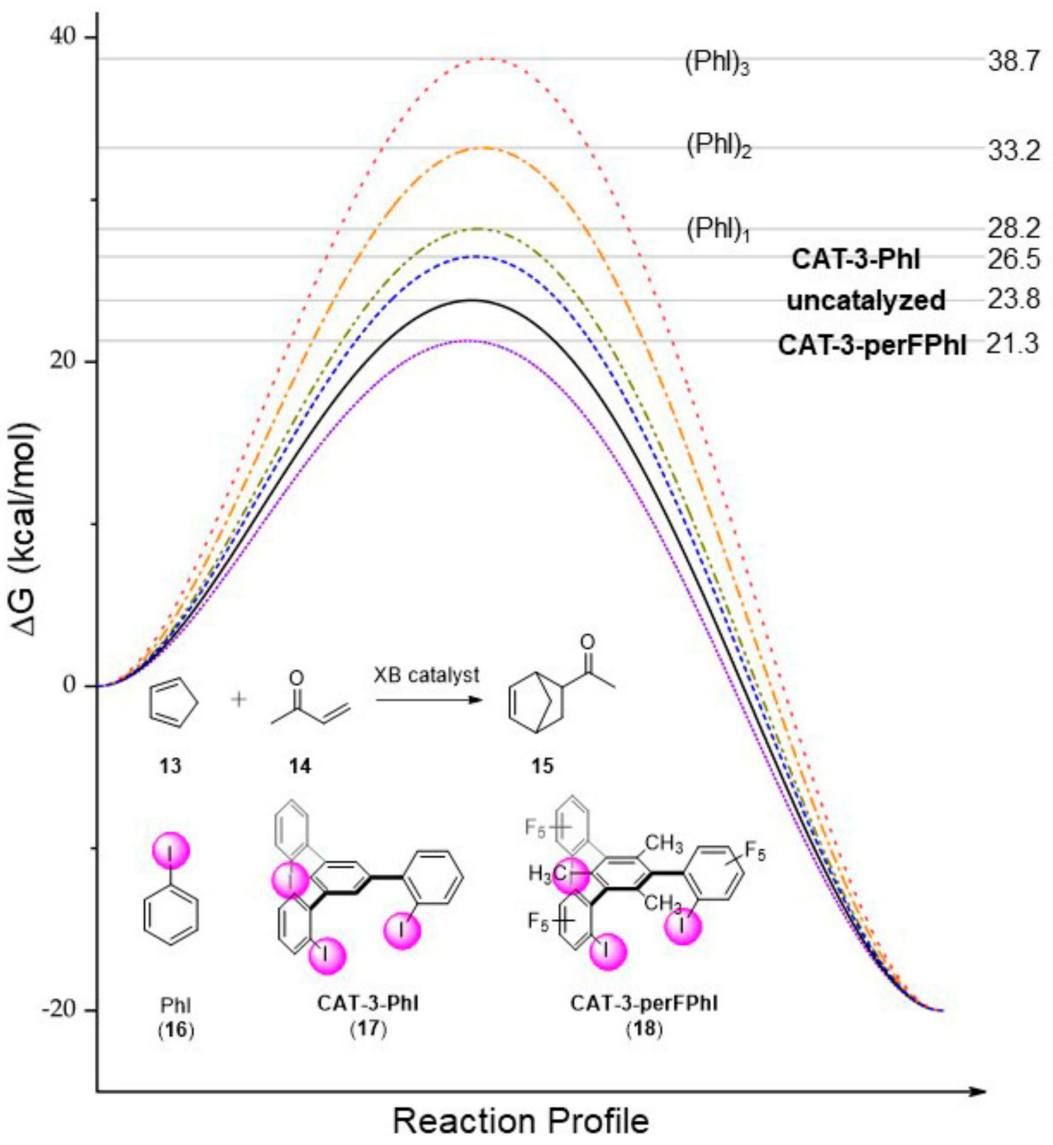
Calculated reaction barriers for uncatalyzed and XB-catalyzed Diels–Alder reactions.

Firstly, Diels–Alder addition between cyclopentadiene (**13**) and buten-2-one (**14**) was studied. The effect of increasing the number of XBs on the reaction activation free energy was examined, by adding one more iodobenzene molecule systematically as the catalytic system (up to three iodobenzene molecules). It was observed that the activation enthalpy is decreased by about 4–5 kcal/mol for each addition of iodobenzene, in line with the increased number of XBs. However, bringing together catalyst and substrates in the tightly bound TS leads to a notable decrease in entropy, the effect of which cannot be ignored. Indeed, the activation free energy increases by 5 kcal/mol for each addition of iodobenzene (Figure 4).

**Figure 5:**
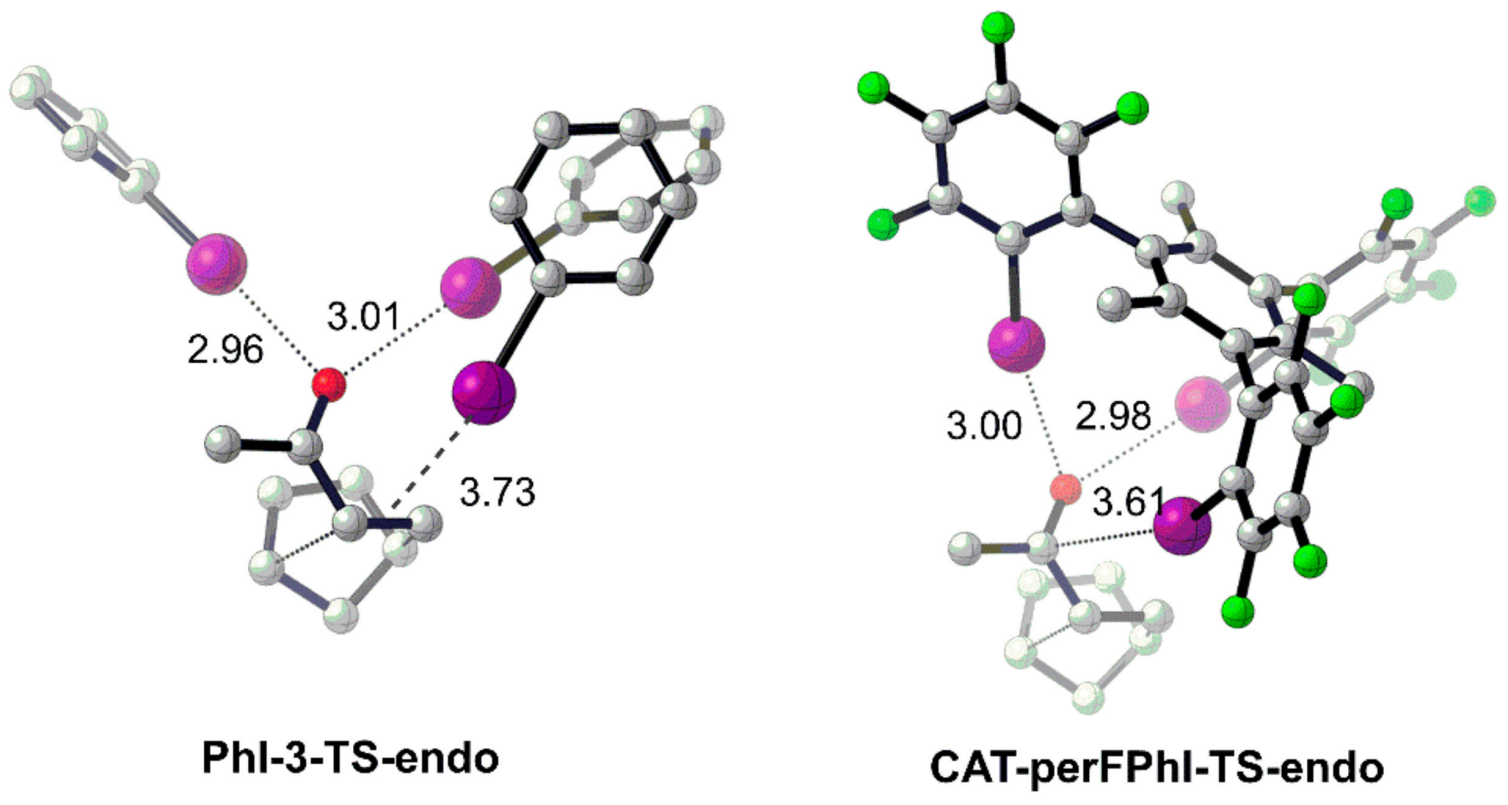
Optimized transition states, PhI-3-TS-endo and CAT-3-perFPhI-TS-endo, showing the similar halogen bond lengths. Interaction distances are shown in Å. Hydrogen atoms are omitted for clarity.

One plausible strategy to overcome the unfavorable entropy effect is to design a molecule with a suitable geometric arrangement that could form three XBs at the same time. The unfavorable entropy contribution would be paid for in the synthesis stage when the three donors are brought together. Accordingly, a tripodal catalyst CAT-3-perFPhI (**18**) was designed, incorporating three fluorinated iodobenzene moieties (Figure 4). When the test Diels–Alder reaction was calculated for CAT-3-perFPhI, a lowered reaction free energy barrier of 21.3 kcal/mol was obtained, compared to 23.8 kcal/mol for the uncatalyzed reaction, indicating the success of the design introducing multiple XBs simultaneously to reduce the entropy penalty. Importantly, the interaction distances of three XBs observed in CAT-3-perFPhI-TS-endo, measured at 2.98, 3.00, and 3.61 Å, are very close to the corresponding distances in PhI-3-TS-endo, at 2.96, 3.01, and 3.73 Å (Figure 5). The XB strengths in CAT-3-perFPhI-TS-endo were estimated using a fragment-based approach to be 6.6, 6.2, and 4.1 kcal/mol, respectively.

The reaction kinetics was also assessed for the uncatalyzed and CAT-3-perFPhI-catalyzed Diels–Alder reaction, using the energetic span model of Shaik and Kozuch [72,73,74]. It was found that the computed turnover frequency (TOF) for the catalyzed reaction increases with decreasing temperature, while, for the uncatalyzed reaction, the rate constant decreases with decreasing temperature. For instance, upon lowering the temperature from 298.15 to 248.15 K, TOF for the catalyzed reaction increases from 5.5 h^−1^ to 28 h^−1^; for the uncatalyzed reaction, the rate constant decreases from 5.7 × 10^−2^ to 1.9 × 10^−3^ M·h^−1^.

Results from SMD modeling of common organic solvents chloroform and *n*-hexane showed that free energy of activation for both uncatalyzed and CAT-3-perFPhI-catalyzed reactions increases compared to the gas-phase results, and the catalyzed reaction is still faster in both solvents.

Catalysis by CAT-3-perFPhI of a Claisen rearrangement reaction was also studied (Table 2). In *n*-hexane, the catalyzed reaction was predicted to have a slightly higher reaction barrier of 28.3 kcal/mol at 318 K than 26.5 kcal/mol for the uncatalyzed reaction, but TOF of the catalyzed reaction is in the same order of magnitude as the kK_eq_ of the uncatalyzed reaction. TOF calculations showed that, when the temperature is lowered to 273 K, TOF of the catalyzed reaction becomes two orders of magnitude higher than kK_eq_ of the uncatalyzed reaction, allowing for catalytic Claisen rearrangement by CAT-3-perFPhI.

It should be noted that, when compared to the similar and well-studied H-bond catalysis [75,76,77,78,79,80], there are far fewer studies of catalysts design based on XB. The larger sizes of halogen atoms and the more stringent structural requirement of XB make the design of multidentate XB catalysts more challenging. Despite being able to form three XBs simultaneously, the designed neutral tridentate catalyst CAT-3-perFPhI (**18**) only lowers the reaction barrier of the benchmark Diels–Alder reaction by 2.5 kcal/mol (Figure 4). This is to be compared to the bidentate cationic catalyst developed by Huber’s group, which lowers the reaction barrier of the same reaction by 3.0 kcal/mol (Figure 2a) [52], in agreement with experimental observation that cationic XB catalysts are more potent than neutral ones. However, as noted in Section 3.1, there are several advantages of neutral XB catalysts, which still make them an attractive target for catalyst design. As of today, the designed catalyst is yet to be synthesized and verified experimentally.

#### 3.2.3. I_2_ as an XB Catalyst in Organic Reactions

In recent years, much effort was devoted to computational and experimental studies of the mechanism of I_2_-catalyzed reactions. By far, the most comprehensive computational mechanistic study was carried out by the Breugst group [81], who investigated the mechanism of four different organic transformations apparently catalyzed by molecular iodine at the B2PLYP-D3/aug-cc-pVTZ/IEFPCM//M06-2X-D3/6-311+G(d,p)/IEFPCM level, with the aug-cc-pVTZ-pp basis set for iodine atoms.

**Figure 6:**
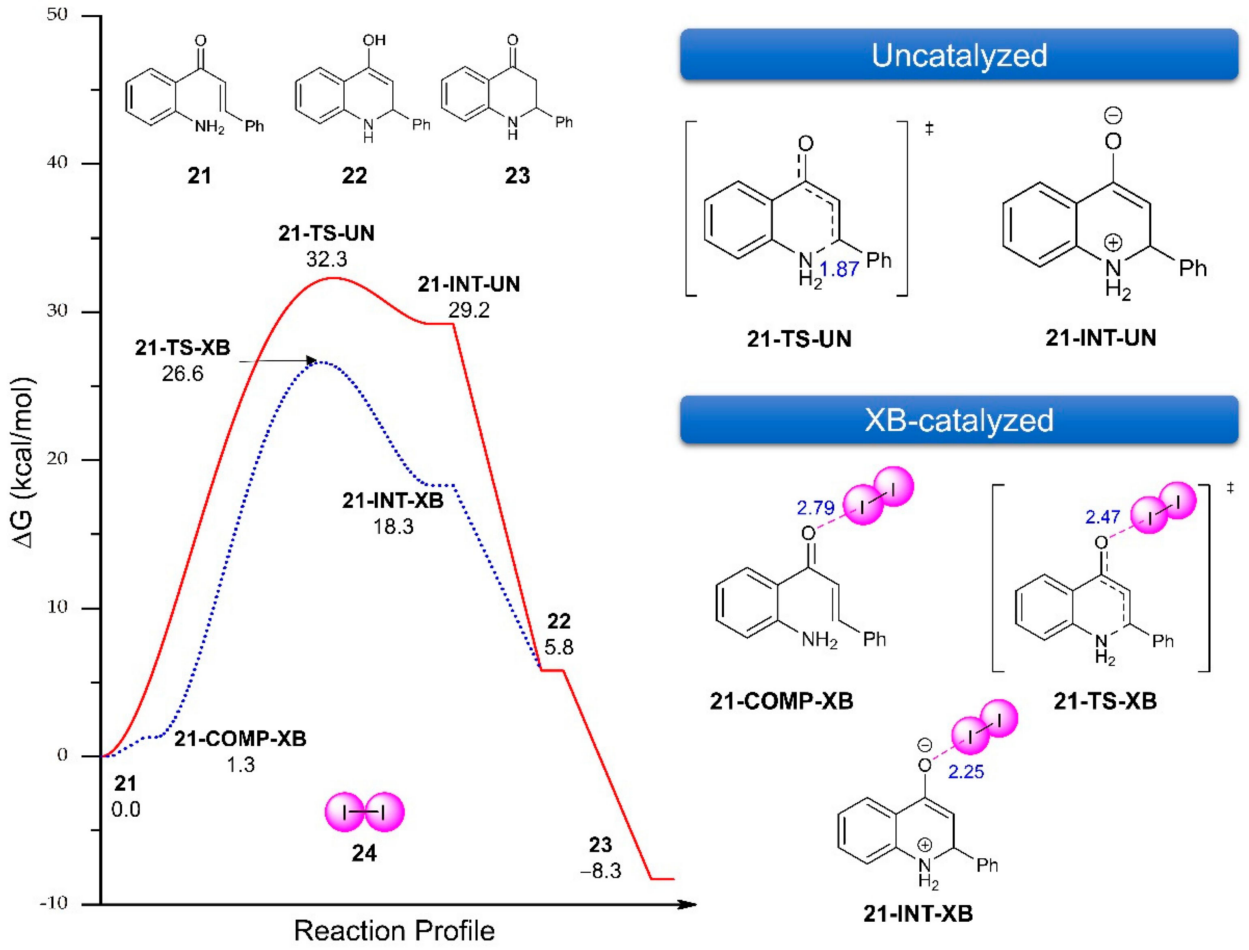
Uncatalyzed (red solid line) and I_2_-calculated (blue dotted line) reaction profiles for intramolecular cyclization of aminochalcone (**21**).

The first reaction studied was intramolecular cyclization of aminochalcone (Figure 6). The mechanism of the uncatalyzed reaction was explored initially, and the free energy barrier of the intramolecular nucleophilic addition of the aniline moiety on the Michael acceptor was determined to be rather high at 32.3 kcal/mol, explaining why the reaction does not occur without a catalyst. Subsequent proton transfer and tautomerization furnish the product.

In the I_2_-catalyzed cyclization, the free energy barrier of the nucleophilic addition step is lowered by 5.7 kcal/mol compared to the uncatalyzed reaction of 26.6 kcal/mol. The authors attributed this to the stabilization of the developing partial negative charge on the Michael acceptor by a very short halogen bond to I_2_, measured at 2.47 Å.

**Figure 7:**
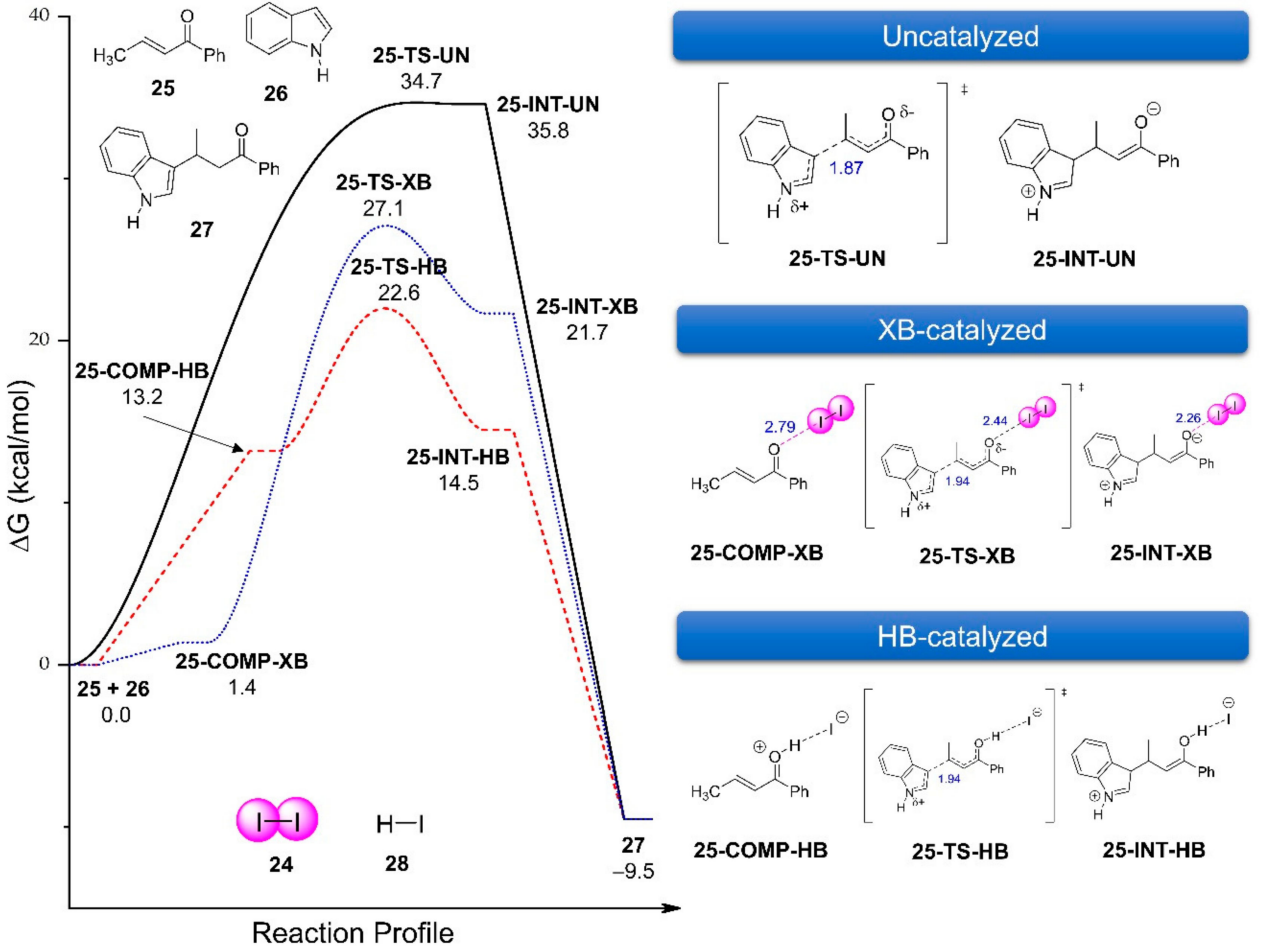
Calculated reaction profiles of uncatalyzed (black solid line), I_2_ (blue dotted line), and HI-catalyzed (red dashed line) intermolecular Michael addition of indole to *trans*-crotonophenone (**25**).

Similarly, I_2_ was found to lower the free energy barrier of intermolecular Michael addition of pyrrolidine to methyl acrylate, from 18.3 kcal/mol of the uncatalyzed reaction to 15.9 kcal/mol, of intermolecular Michael addition of methyl pyrrole to nitrostyrene from 26.6 kcal/mol to 24.7 kcal/mol, and of intermolecular Michael addition of indole to *trans*-crotonophenone from 34.7 kcal/mol to 27.1 kcal/mol (Figure 7).

**Figure 8:**
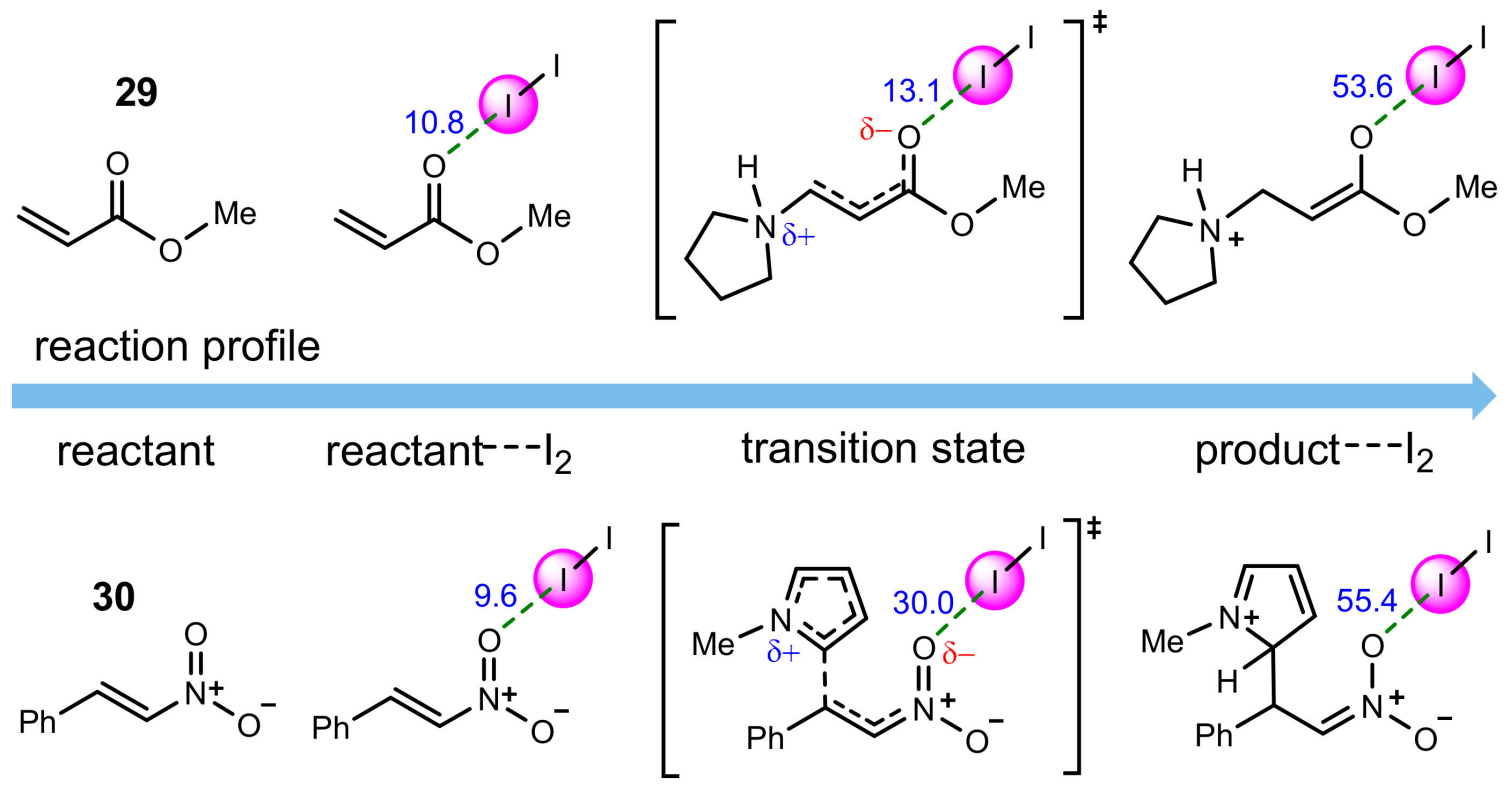
NBO population analysis of XB interaction energies for selected reactions.

Analysis of the halogen bonding interaction of various species of two selected reactions by natural bond orbital (NBO) population analysis [82] showed that XB interaction energies increase along the reaction profile, in line with the developing partial negative charges on corresponding XB-donors. The interaction energies could reach a remarkable magnitude of more than 50 kcal/mol in the zwitterionic intermediates (Figure 8).

One other possible mechanistic pathway, namely, hidden Brønsted acid catalysis, was also investigated. Several possible pathways to generate hydroiodic acid (HI) were calculated but were found to be highly endergonic. Nevertheless, the authors calculated HI-catalyzed addition of indole to *trans*-crotonophenone (Figure 7) and found that HI is also an excellent catalyst for this reaction. The reaction barrier is even slightly lower than the I_2_-catalyzed pathway, with 22.6 kcal/mol for the former versus 27.1 kcal/mol for the latter. To help determining the reaction mechanism, the authors carried out careful experiments to directly compare the reaction rates of I_2_ and HI-catalyzed reactions in acetonitrile. Results showed that I_2_ gave on average 72% ± 3% yield in 3 min while HI gave only 40% ± 3% yield for the same duration, in support of the XB catalysis mechanism. In 2017, the same group performed a detailed experimental investigation and, based on their new experimental result, they ruled out the Brønsted acid catalysis in favor of the XB catalysis mechanism [33].

#### 3.2.4. I_2_-Catalyzed *iso*-Nazarov Cyclization of Conjugated Dienals

The XB-catalyzed Nazarov cyclization attracted much attention recently [54,83,84]. Breugst and co-workers reported a combined experimental and computational study of I_2_-catalyzed Nazarov cyclization [84]. Based on their results of kinetic studies, i.e., the reaction being first order in I_2_, and the reaction barrier, as well as comparative control studies, the authors could rule out the Brønsted acid pathway in favor of the XB pathway. Their calculated activation barrier of the XB pathway (84 kJ/mol) is in close agreement with the experimental determined value (ΔG^≠^_298_ = 86.8 kJ/mol). The reaction profile and transition state structure are similar to those presented in Figure 6.

**Figure 9:**
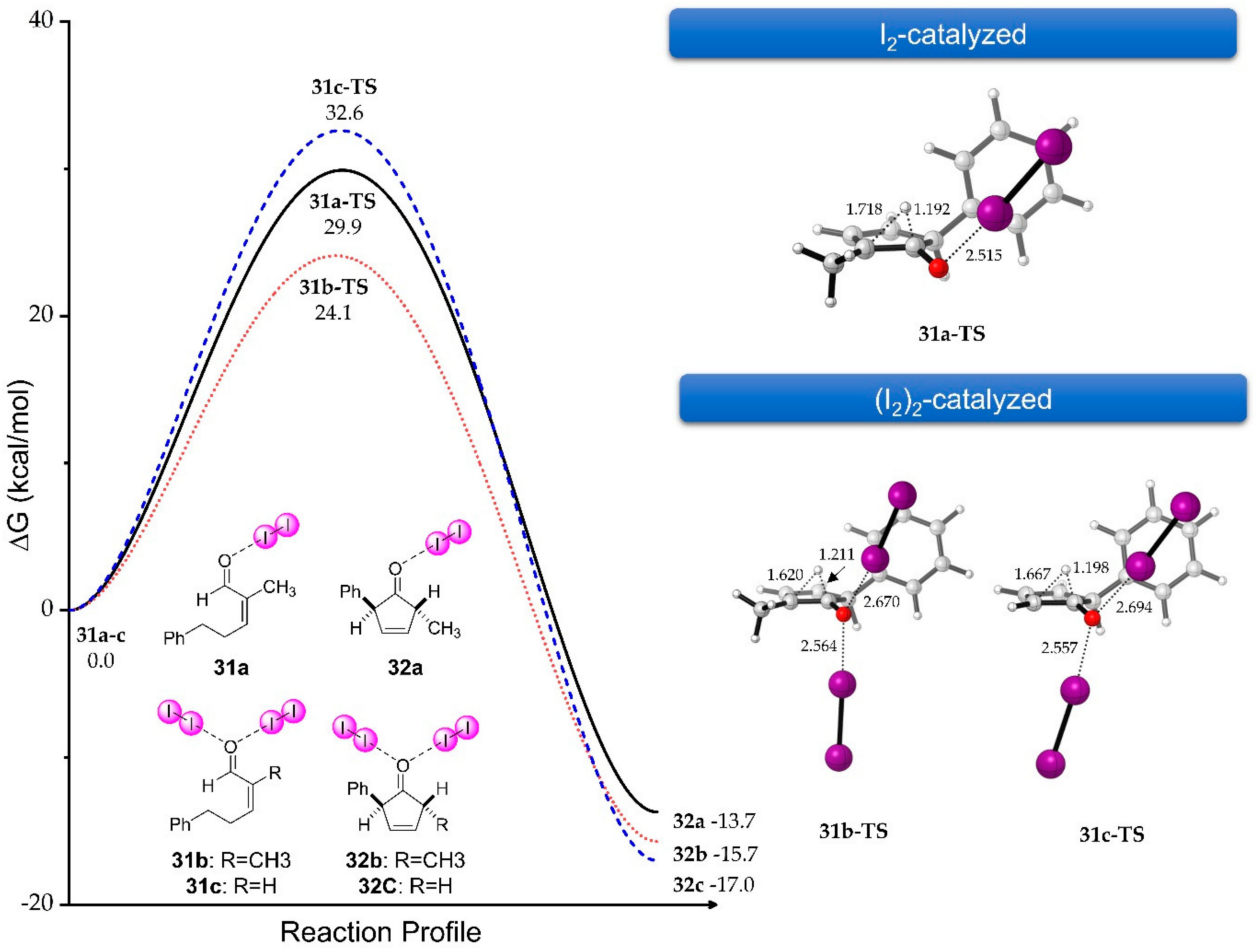
Calculated reaction profiles for the cyclization step of I_2_-catalyzed *iso*-Nazarov cyclization of conjugated dienals. Interaction distances are given in Å.

Riveira and coworkers recently studied a similar I_2_-catalyzed *iso*-Nazarov cyclization of conjugated dienals at the PCM (ethyl acetate)/M06-2X/6-311+G(d,p) (aug-cc-pVTZ-PP basis set for I) level (Figure 9) [83]. The reaction barrier (ΔG^≠^_393_) of I_2_-catalyzed cyclization of **31a** was computed to be quite high at 29.9 kcal/mol, although it is lower than the barrier of the uncatalyzed reaction at 38.1 kcal/mol. The authors then proposed a new catalytic pathway involving two I_2_ molecules in the cyclization step TS, namely, **31b-TS** (Figure 9). The reaction barrier was further reduced by 5.8 kcal/mol to 24.1 kcal/mol. In further support of this proposed pathway, the reaction barrier for the substrate of **31c** (R = H) was computed to be very high at 32.6 kcal/mol, in line with the experimental finding where only traces amount of product could be obtained for this substrate.

It should be noted that the reference used to calculate reaction barriers is the XB-bonded pre-TS complexes rather than the free catalyst and substrates. The complexation process of catalysts and substrates via halogen bond is not necessarily favorable in terms of free energy [81]. Use of binding complexes as an energy reference might lead to an inaccurate comparison between pathways that differ in molecularity, resulting in a bias favoring higher-molecularity pathways. As pointed out in our early discussion, Breugst et al. determined the reaction order with respect to molecular iodine for a similar Nazarov cyclization reaction to be one.

#### 3.2.5. Dihalogen-Catalyzed Michael Addition Reactions

By far, the majority of computational and experimental studies of XB catalysis focused on the element iodine. In 2019, Bickelhaupt and co-workers reported a comprehensive computational study of aza-Michael addition of pyrrolidine to methyl acrylate catalyzed by dihalogen molecules (X_2_), namely, F_2_, Cl_2_, Br_2_ and I_2_ [49]. All geometries were optimized and characterized at the M06-2X/def2-TZVP level. Scalar relativistic effects were accounted for using the zeroth-order regular approximation (ZORA) [85,86]. DLPNO-CCSD(T) calculations were performed using the def2-TZVP basis set on M06-2X/def2-TZVP geometries.

**Figure 10:**
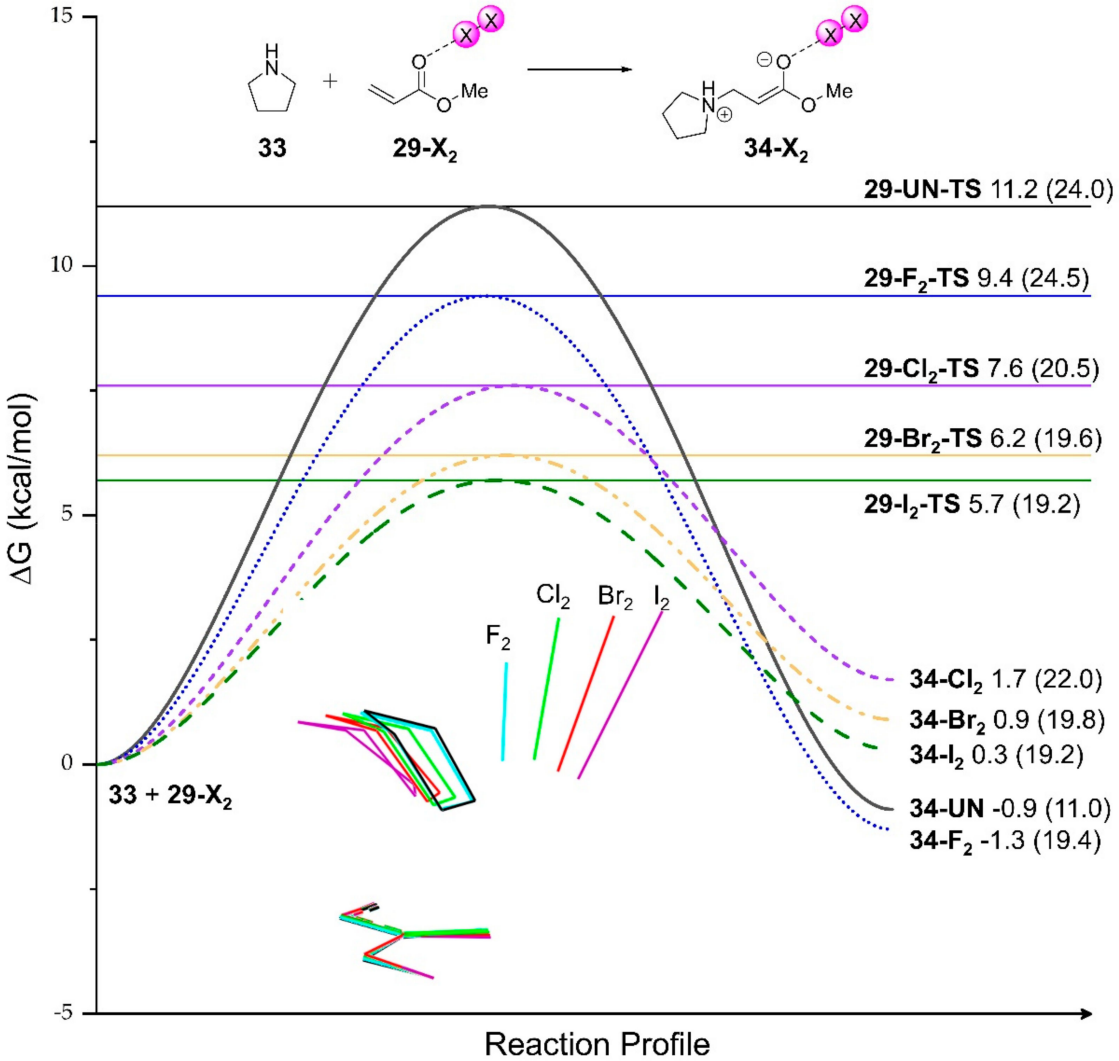
Calculated reaction profiles for the C–C bond addition step of the uncatalyzed and dihalogen (X_2_)-catalyzed Michael addition reactions, at the M06-2X/def2-TZVP level. Reaction energies are reported in kcal/mol and free energies at 298.15 K and 1 atm are given in parentheses. Overlaid transition state (TS) structures are shown at the bottom and colored as follows: uncatalyzed black, F_2_ cyan, Cl_2_ green, Br_2_ red, and I_2_ purple.

The authors calculated the four dihalogen molecule-catalyzed reactions at the M06-2X/def2-TZVP level and compared them to the uncatalyzed reaction (Figure 10) [49]. They observed a systematic decrease of the reaction barrier down Group 17, from 9.4 kcal/mol for F_2_ to 5.7 kcal/mol for I_2_. In comparison, the uncatalyzed reaction has an activation barrier of 11.2 kcal/mol. Correspondingly, the TSs are reached earlier and the formed C–N bonds are longer going down the group. Overlaid TSs (Figure 10) clearly show a systematic change of the TS structures. These observed trends are consistent with the experimental observation of the trend of activity within the halogen group.

**Figure 11:**
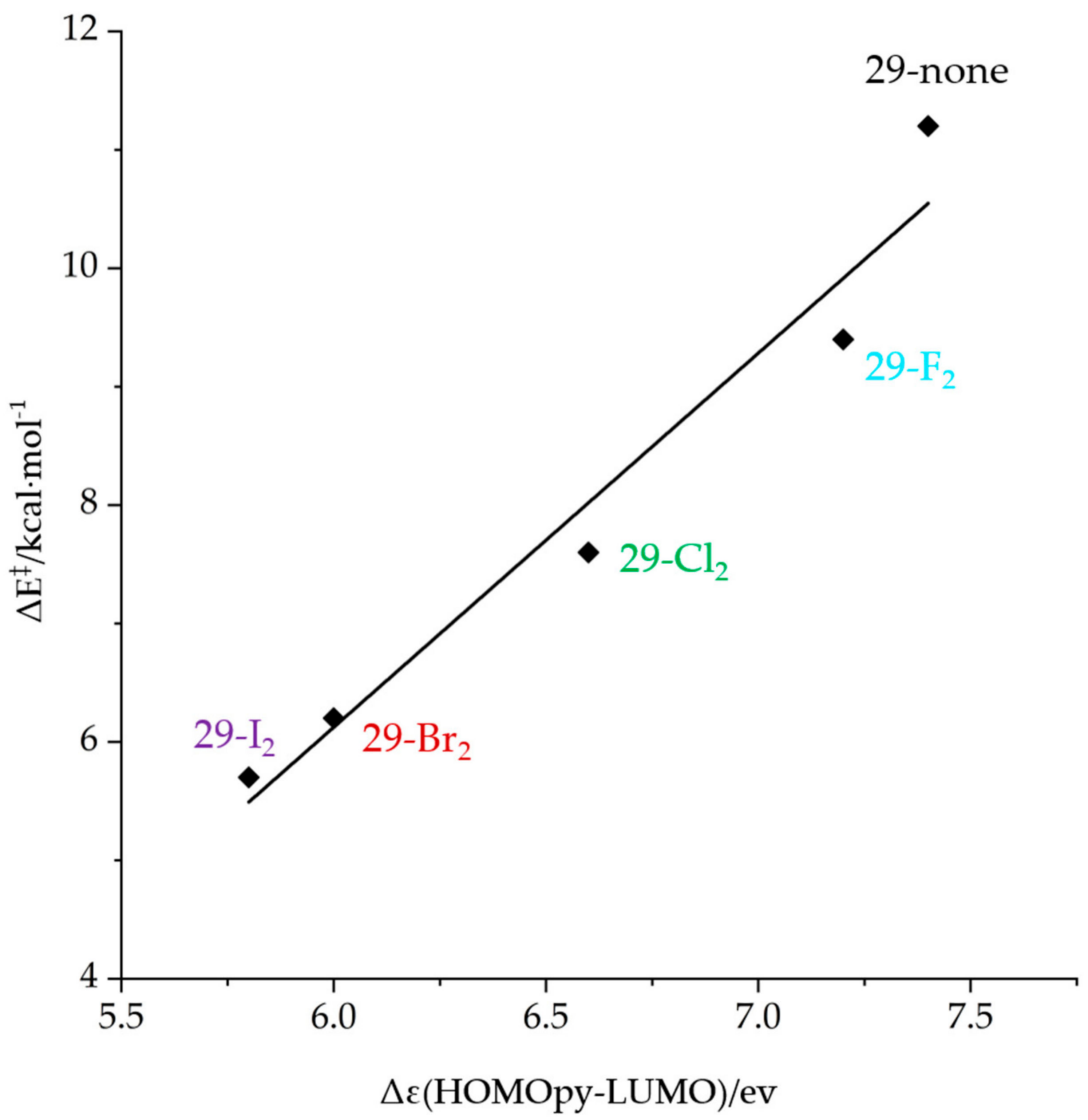
Calculated activation barriers for Michael addition reactions catalyzed by dihalogens versus the HOMO_py_–LUMO gap Δε, at the M06-2X/def2-TZVP level.

The activation of substrates by XB interaction was commonly thought to originate from lowering the LUMO energies of the electrophiles and, hence, stronger HOMO (nucleophile)–LUMO (electrophile) interaction. Indeed, a good correlation (*R^2^* = 0.97) was obtained between reaction barriers and HOMO–LUMO gaps (Figure 11). However, when the interaction was analyzed using the activation strain model (ASM) [87], the authors found unexpectedly that the physical factor controlling the computed reactivity trend is not the abovementioned enhanced donor–acceptor interaction, but rather a diminished Pauli repulsion between the lone pair of the nucleophile pyrrolidine and the Michael acceptor’s π-electron system. We refer interested readers to the original paper [49] for more in-depth discussion of the energy decomposition analysis, which is beyond the scope of the current review.

Understanding the origin of halogen bond activation is important in many aspects of XB catalysis, particularly the design of new catalysts. Thus, this new view could potentially change the way we understand XB catalysis and design new XB catalysts. However, several aspects of the computation need further improvement, which is discussed below, and the usefulness of the new interpretation in predicting catalyst activity needs corroboration from other studies.

The molecular structures used in the ASM energy analyses were optimized in the gas phase, which is not ideal for the nucleophilic addition reaction studied here since the addition product is a zwitterionic intermediate. In comparison, Breugst and co-workers reported a very different free energy reaction profile for the same I_2_-catalyzed Michael addition reaction, calculated using the SMD solvent model [81], from the one presented in Figure 10. For example, in Figure 10, I_2_-INT has the same energy as I_2_-TS, ΔG_298_ = 19.2 kcal/mol, whereas Breugst calculated an energy difference of 6.5 kcal/mol for the same two species in the presence of dichloromethane solvent. Unless the solvent effect is considered, which is certainly critical for accurate geometries and energies for catalytic reactions happening in solution, the gas-phase analysis might have limited relevance to experimental XB catalysis.

Similar to the I_2_-catalyzed *iso*-Nazarov cyclization reaction discussed in Section 3.2.4, reaction barriers were calculated with reference to the halogen bonded complexes. This could lead to potential bias toward catalyzed reactions. Furthermore, we are yet to see experimental studies employing F_2_ or Cl_2_ as a catalyst in organic reactions that involve reactive compounds such as pyrrolidine.

#### 3.2.6. XB in Metal Acetate-Catalyzed Halolactonization

**Scheme 3:**
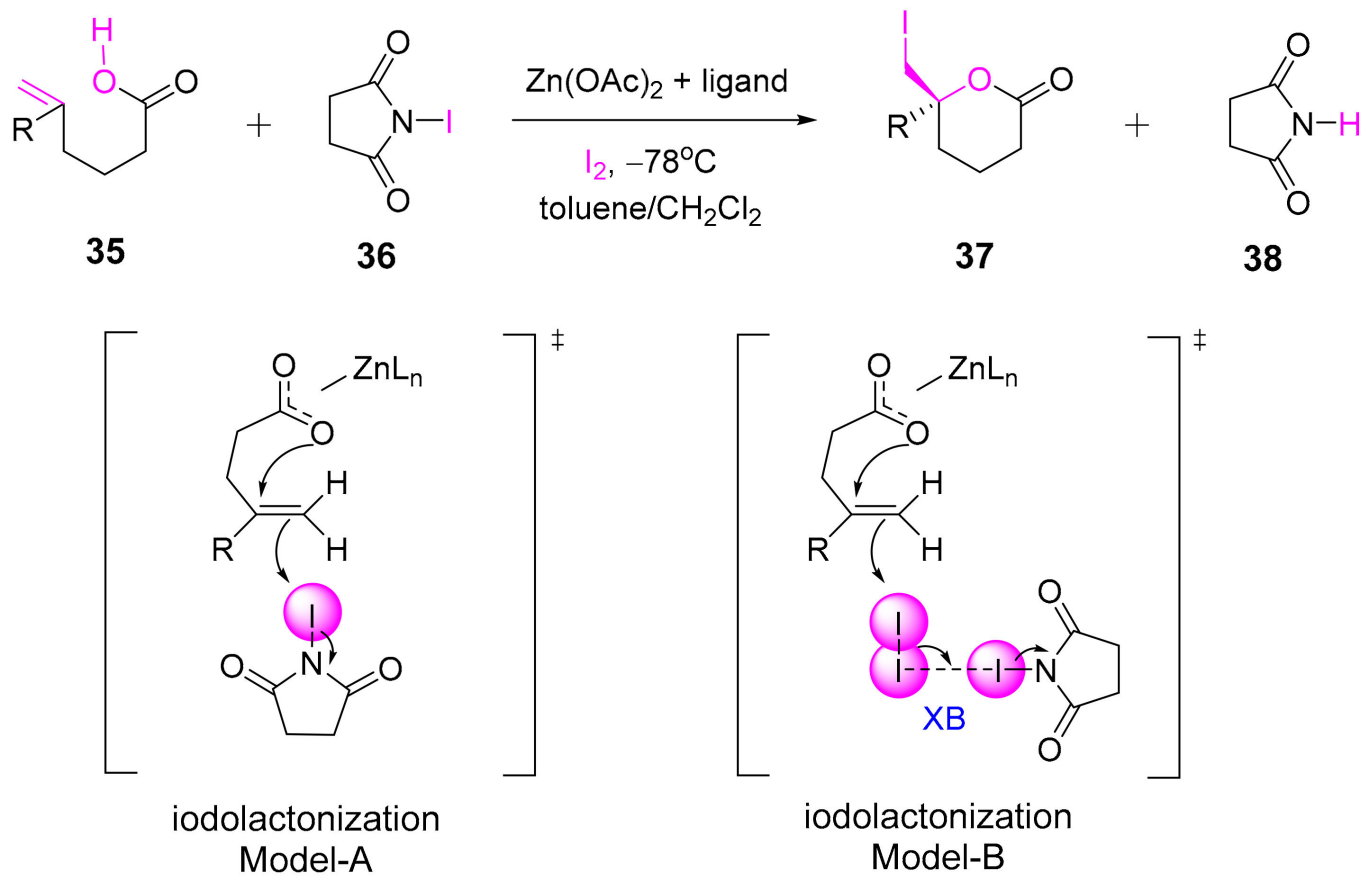
Zinc acetate-catalyzed iodolactonization reaction of allyl acetic acid (**35**).

The above examples are computational studies of neutral XB-catalyzed reactions. There are many literature reports of halogenation reactions where, prior to the halogenation step, halogen bonds are formed between some Lewis base and the halogenation agents [88,89,90]. Such XBs are transient in nature, as the X–A (X is the halogen and A is some heteroatom) bonds are cleaved in TSs. Recently, Arai and co-workers reported a metal acetate-catalyzed asymmetric halolactonization, where halogen bonding interaction was proposed as a key secondary interaction in the transition state [91]. Experimentally, the authors found that molecular iodine has a significant impact on the acceleration of the reaction. In their computational study, two TS models were proposed, Model-A and Model-B (Scheme 3). In Model-A, iodination of the double bond occurs directly from *N*-iodosuccinimide (NIS), whereas, in Model-B, it is from I_2_, which simultaneously forms a key XB with NIS. Calculated results showed that the Model-B TS is 12.6 kcal/mol lower in energy than the corresponding Model-A TS, in good agreement with experiments. Molecular iodine and NIS are reactive reagents, and each is susceptible to several plausible reaction pathways. It will require further careful mechanistic studies considering different reaction pathways to confirm the role of XB in this reaction.

### 3.3. Cationic XB Catalysts

Over the past decade, advancements of XB catalysis were largely centered on cationic iodine-containing catalysts based on nitrogen heterocycle scaffolds, e.g., imidazoliniums, imidazoliums, and triazoliums, which were reported to catalyze a variety of organic transformations.

#### 3.3.1. XB-Catalyzed Reduction of Quinolines by Hantzsch Ester

**Scheme 4:**
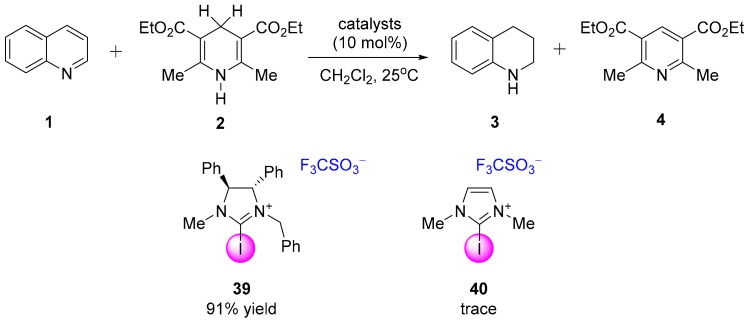
Reduction of quinoline by Hantzsch ester catalyzed by imidazolinium and imidazolium.

Reduction of quinoline by Hantzsch ester is an important organic reaction that was explored many times in the literature as a benchmark reaction for new XB catalysts [16,21,26]. In 2014, Tan et al. reported that iodoimidazolinium compound (**39**) (Scheme 4) can catalyze the reduction of quinoline by Hantzsch ester to give 91% yield in dichloromethane at room temperature. Interestingly, a related iodoimidazolium catalyst (**40**) gave only a trace amount of product yield. Recently, our group reported a comprehensive computational mechanistic study of the reaction at the M06-2X/6-311+G(d,p)(def2-TZVPD for iodine)//M06-2X/6-31G(d)(def2-SVP for iodine) level, with the SMD solvent model and SMD18 correction [43]. For all calculations involving **39** and **40**, the triflate anion was included to neutralize the catalytic systems.

**Figure 12:**
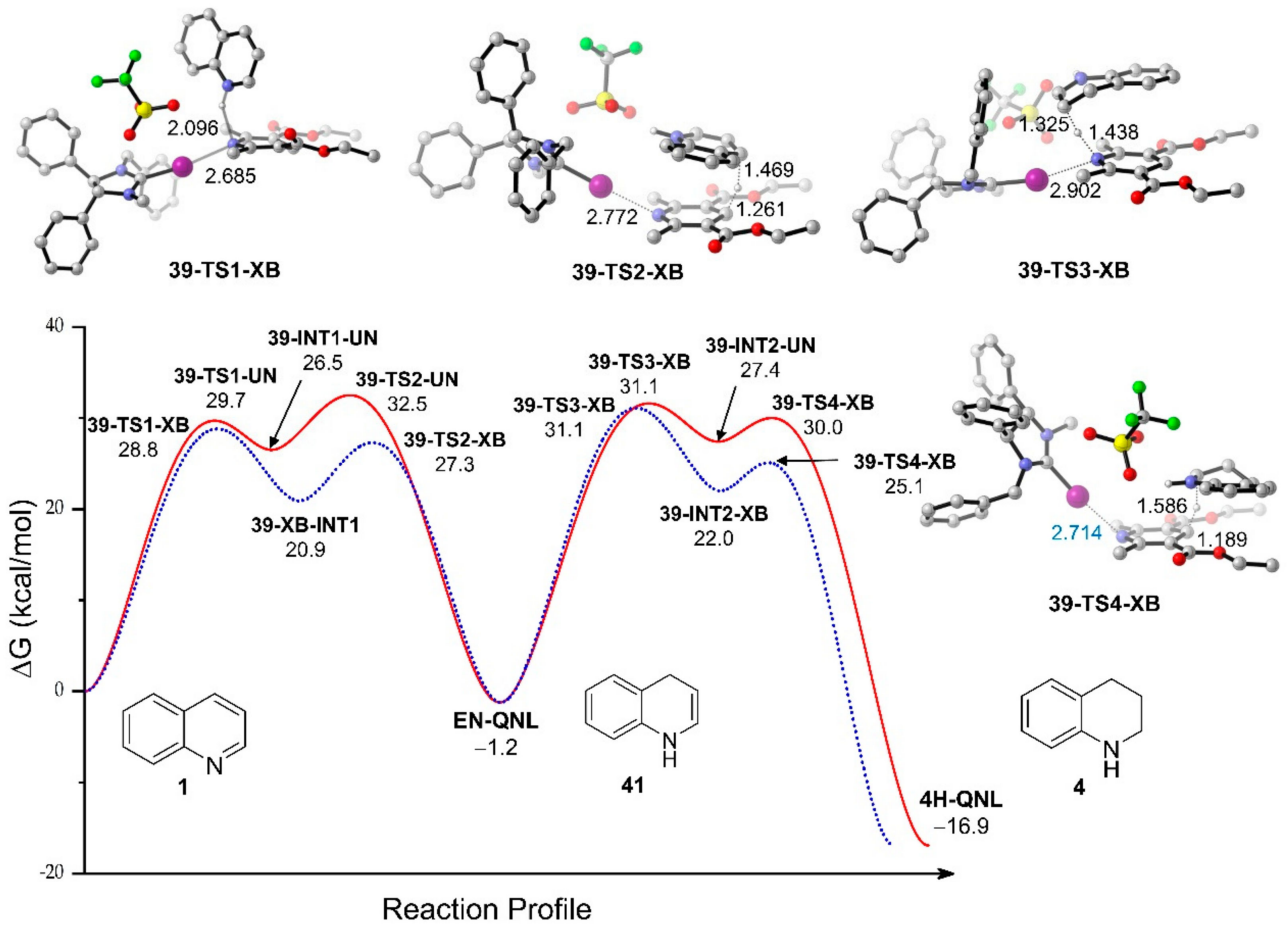
Calculated reaction profiles for the uncatalyzed (red solid line) and halogen bond-catalyzed (blue dotted line) pathways. Distances are in Å and A–I···B angles are in degrees. C–H hydrogen atoms except for the transferring hydride or proton are omitted for clarity.

Similarly, the mechanism of the XB-catalyzed reaction pathway was investigated. The lowest energy pathway is presented in Figure 12 and compared to the uncatalyzed pathway. One major finding is that it is preferable for the iodine atom of **39** to bind to the deprotonated nitrogen atom of Hantzsch ester in the key hydride transfer transition states, namely, **39**-TS2-XB and **39**-TS4-XB, instead of the nitrogen atom of quinoline [21]. Compared to the uncatalyzed reactions, XBs lower the reaction free energy barriers of hydride transfer steps by about 5 kcal/mol. However, they do not appreciably lower the barriers of the proton transfer steps.

**Figure 13:**
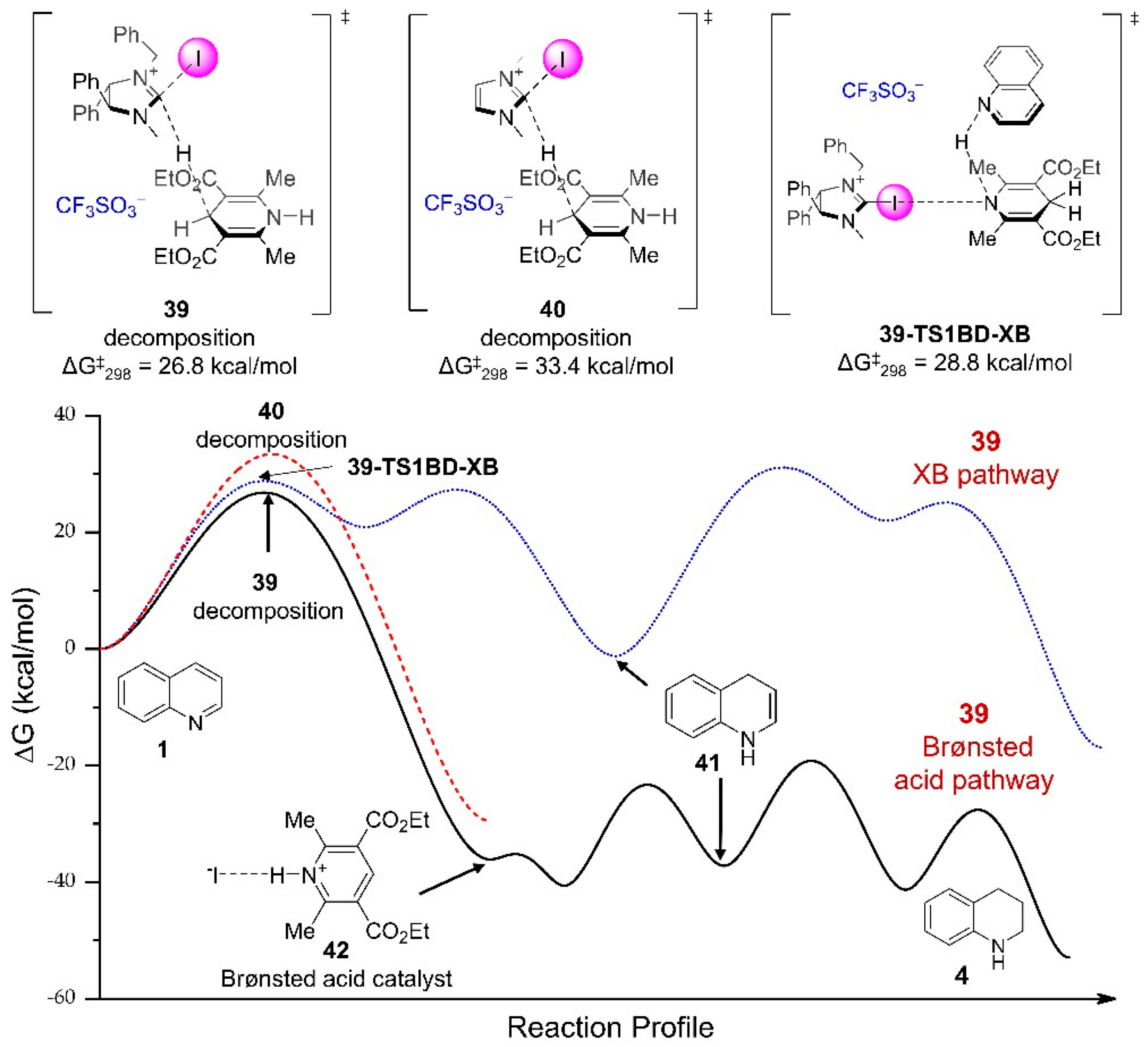
Calculated pathways for Brønsted acid-catalyzed (black solid line) and XB-catalyzed (blue dotted line) reduction of quinoline by **39**. For comparison, the decomposition of **40** to generate Brønsted acid catalyst is shown in red dashed line.

More significantly, the report is the first one of which we are aware that discovered a Brønsted acid pathway (Figure 13) that was predicted to be competitive to the hypothesized XB catalysis. Hantzsch ester, being a reductant, can reduce the charged iodoimidazolinium moiety of **39** to generate a Brønsted acid with a relatively low barrier of 26.8 kcal/mol, which is lower than that of the first transition state of the XB catalysis pathway (28.8 kcal/mol). Following the generation of acid catalyst, the reduction can be catalyzed very efficiently. In comparison, when reduction of **40** by Hantzsch ester was calculated, the barrier was higher at 33.4 kcal/mol, reflecting the aromatic nature of the imidazolium core of **40**. The result is in good agreement with the experimental finding where **40** failed to catalyze the reaction (Scheme 4).

This computational study highlights the necessity and challenges of comparing XB to other possible pathways, particularly the Brønsted acid catalysis pathway. Unlike the XB pathway, the Brønsted acid pathway requires the acid to be generated before the catalysis initiates, which may be accomplished in many unexpected ways and from many overlooked sources. Future computational mechanistic studies should take precaution to avoid too narrow a consideration of the Brønsted acid pathway in favor of the XB pathway. The view is echoed by a recent experimental report of the Scheidt group [28], who discovered through control studies that, for cationic XB catalysts, the activity may stem from a trace amount of Brønsted acid produced from adventitious water.

#### 3.3.2. XB-Catalyzed Conjugate Addition of Thiophenes to Enones and Enals

**Figure 14:**
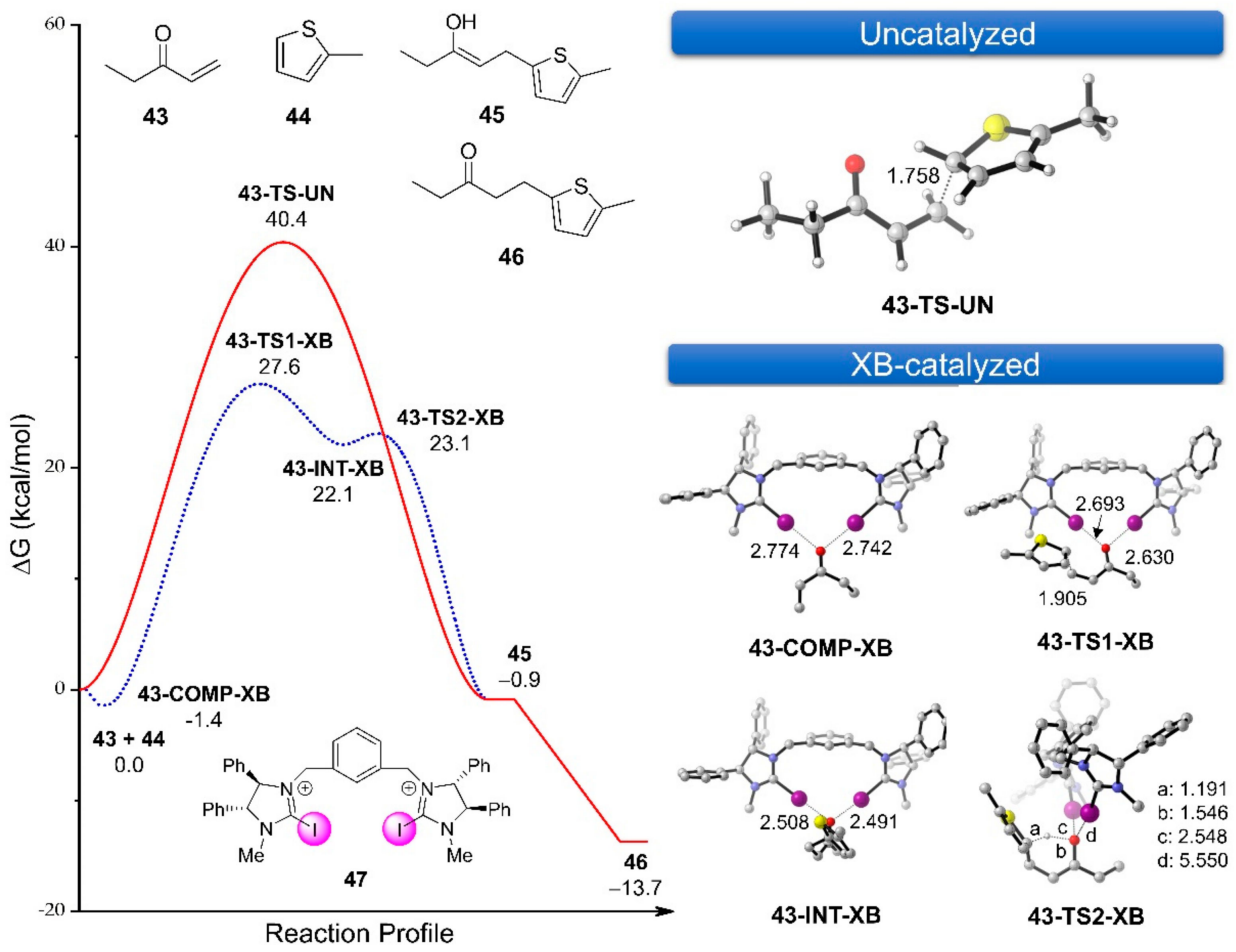
Calculated reaction profiles for uncatalyzed (red solid line) and iodoimidazolinium **47**-catalyzed (blue dotted line) conjugate addition of methylthiophene to enone.

Tan et al. reported an XB-catalyzed conjugate addition of thiophenes to enones and enals [92]. A bidentate imidazolinium-based catalyst (**47**) was found to be the most active catalysts tested. The mechanism of this reaction was investigated by our group using the MN15 functional. The solvent effect was modeled with the SMD method and dichloroethane (DCE) was used as the solvent. Relative free energies at 298 K (ΔG_298_) were reported (Figure 14).

The catalyzed reaction was firstly investigated and it was found to be an asynchronous concerted reaction. The C–C bond formation was immediately followed by a proton transfer, yielding an enol intermediate (**45**), which readily tautomerizes to give the final addition product (**46**). The reaction barrier was calculated to be very high at 40.4 kcal/mol, in accordance with experiment that the reaction does not occur without a catalyst.

In comparison, catalysis by **47** through a pincer type XB binding to the enone substrate changes the reaction mechanism to follow a stepwise pathway (Figure 14). A stable intermediate, **43**-INT-XB, in which catalyst **47** forms two strong XBs to the negatively charged enolate, at 2.51 and 2.49 Å, could be located at a reasonable energy level of 22.1 kcal/mol. The magnitude of XB stabilization in this intermediate was estimated through a distortion-interaction model, assuming the interaction comes mainly from the XBs. A large stabilization energy of 37.8 kcal/mol was calculated and found to be the largest along the reaction profile. This magnitude of stabilization is more than enough to compensate for the entropy loss of forming two XBs simultaneously, which is typically beyond the reach of monodentate XB catalysts except perhaps the strongest ones. The rate-limiting step of the catalyzed reaction was determined to be the first C–C bond-formation step. The reaction free energy barrier was reduced by 12.8 kcal/mol to 27.6 kcal/mol when compared to the uncatalyzed reaction.

The competing Brønsted acid pathway was not investigated computationally. Instead, the experimental result in the same study showed that, in the presence of Cs_2_CO_3_ base, the yield of **46** decreased from 88% to 56%, indicating that a substantial portion of the reaction might go through the Brønsted acid catalysis pathway. However, the source and mechanism of the acid catalyst generation is still unclear.

#### 3.3.3. Catalysis by C–I···π Type of XB Interaction

**Figure 15:**
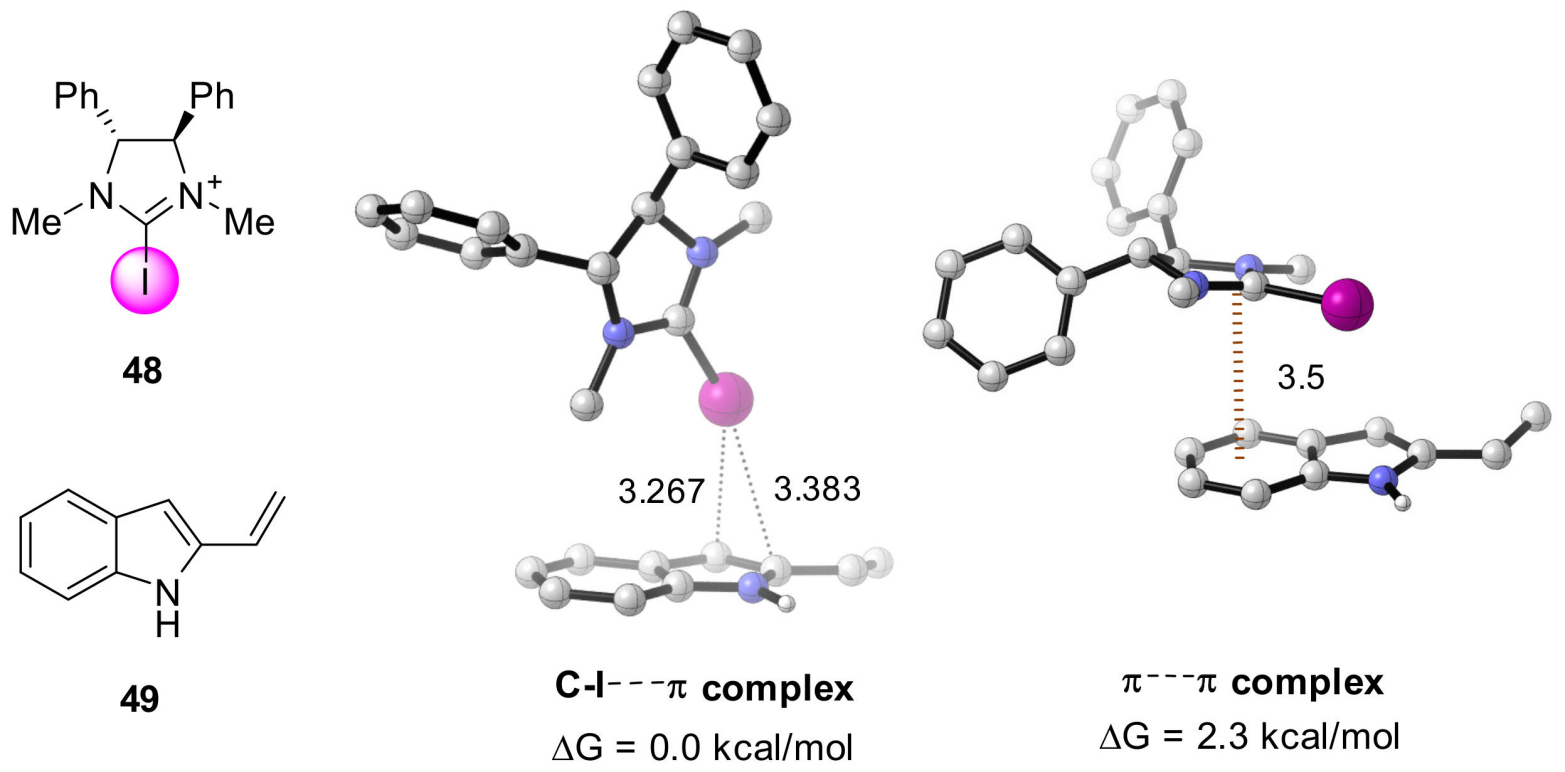
Calculated XB and π···π interaction complexes between **48** and **49**. Interaction distances are in Å and A–I···B angles are in degrees. Hydrogen atoms are omitted for clarity.

Unlike most reported studies of XB catalysis, in which the halogen atoms bind to lone pairs of some heteroatoms, Arai et al. very recently reported the first catalyzed [4+2] cycloaddition whose substrate was proposed to be activated through C–I···π interaction [93]. Calculations done at the M06-2X/6-31G(d) (LANL2DZdp for iodine atoms) level showed that the C–I···π complex between a cationic XB donor (**48**) and 2-alkenylindole (**49**) was 2.3 kcal/mol lower in energy than the π···π complex, which was attributed to the destabilization effect of phenyl moieties of the XB catalyst (Figure 15).

**Figure 16:**
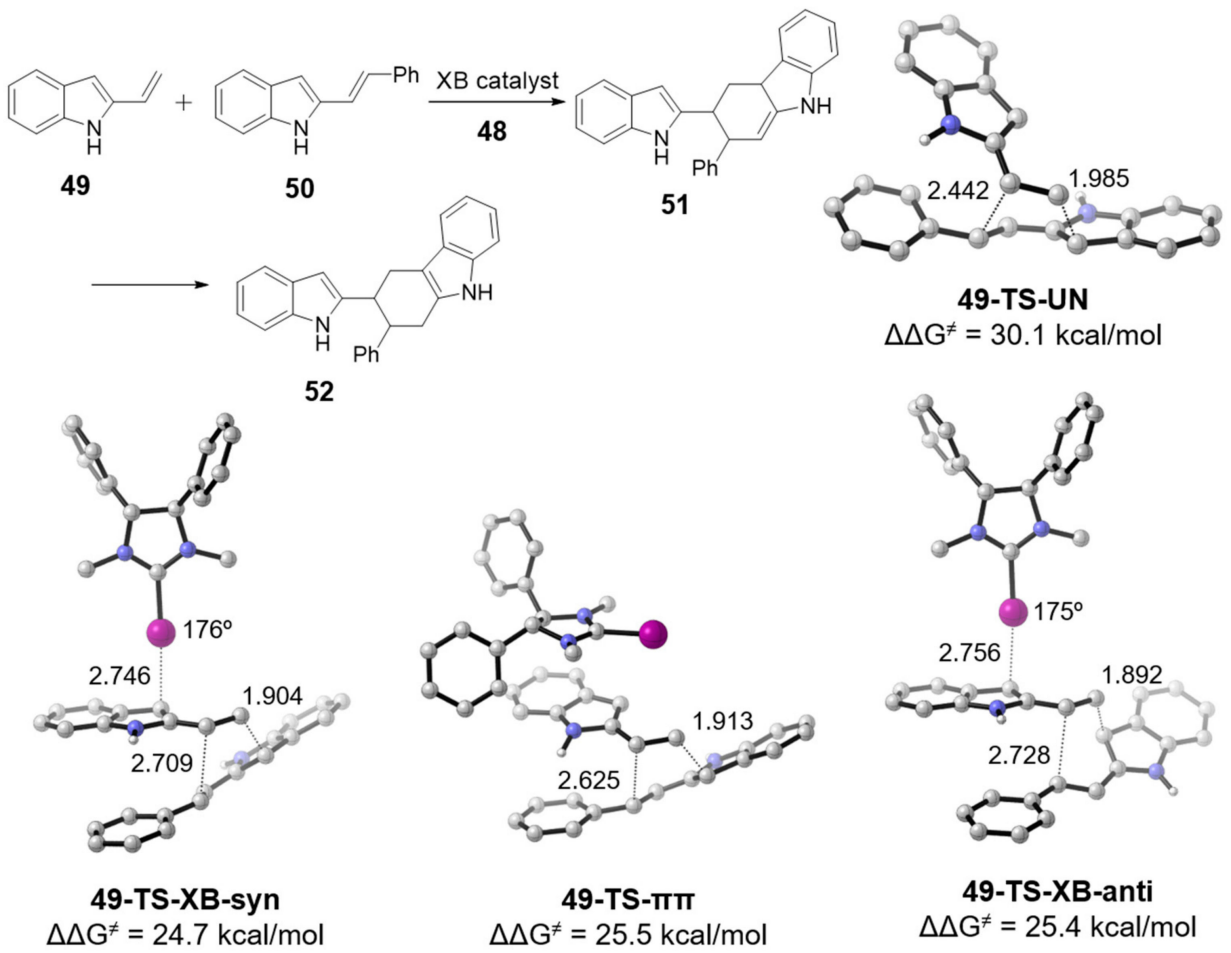
Transition states of [4+2] cycloaddition between indoles. Interaction distances are in Å and A–I···B angles are in degrees. Hydrogen atoms are omitted for clarity.

When the TS catalyzed by C–I···π interaction, namely, **49**-TS-XB-Syn, was compared to the uncatalyzed TS, **49**-TS-UN, the reaction free energy barrier was reduced significantly by 5.4 kcal/mol. Calculations also revealed that the π···π interaction-catalyzed TS, **49**-TS-ππ, was slightly higher in energy, with a reaction barrier of 25.5 kcal/mol (Figure 16). Overall, there is a good agreement between the calculated diastereoselectivity, ΔΔG^≠^ = 0.7 kcal/mol between the *anti* and *syn* TSs, and the experimental results.

The claim of the C–I···π type of XB interaction being able to catalyze an organic reaction in preference to other well-established interactions seems quite promising and may in time lead to more discovery of XB-catalyzed reactions. However, given the scarcity of the reported difference between complexes and TSs of different activation modes, further investigation is needed to establish the C–I···π interaction unambiguously as a new type of XB activation.

### 3.4. Hypervalent Iodine(III)-Catalyzed Reactions

#### ICl_3_-Catalyzed Ring-Opening Polymerization of l-Lactide

Hypervalent iodine(III) compounds are versatile reagents that are used in many organic reactions. The high oxidation state of +3 renders them electrophilic. Therefore, they are able to interact with lone pairs of electronegative atoms to form noncovalent XBs. One of the earliest reports of XB catalysis is the ICl_3_-catalyzed ring-opening polymerization of l-lactide by Coulembier et al. [57]. In 2019, Zeng et al. reported a computational study of the mechanism of this reaction [94].

**Figure 17:**
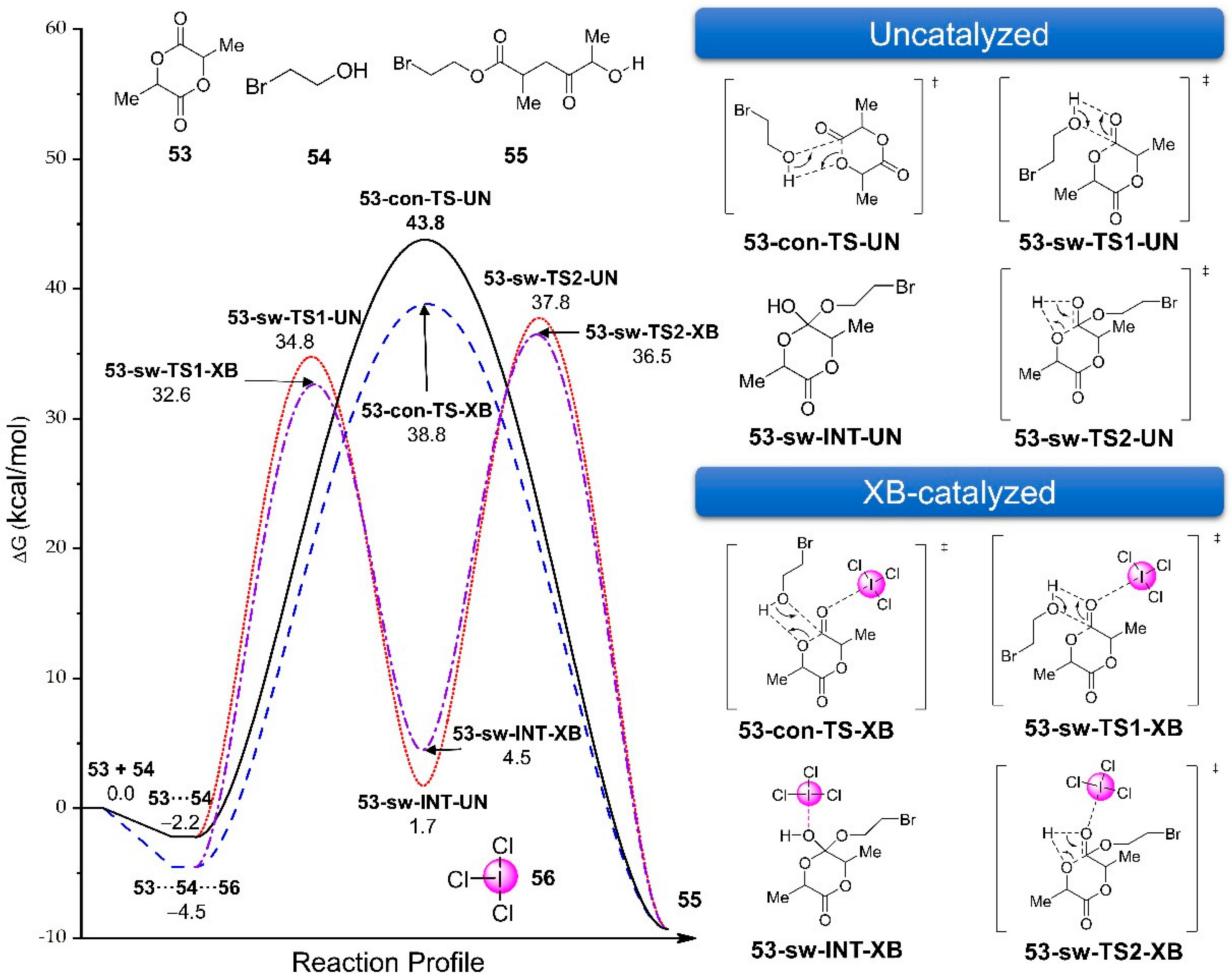
Calculated reaction profiles for the uncatalyzed and ICl_3_-catalyzed ring-opening polymerization of l-lactide. The uncatalyzed concerted pathway is shown as a black solid line, the uncatalyzed stepwise pathway is shown as a red dotted line, the XB-catalyzed concerted pathway is shown as a blue dashed line, and the catalyzed stepwise pathway is shown as a purple dash-dotted line.

Two different pathways, concerted and stepwise, together with the corresponding catalyzed pathways, were investigated at the PBE0/6-311+G(d,p) (aug-cc-pVDZ-PP for iodine) level, and the results are summarized in Figure 17. All calculated pathways have substantially high activation barriers of more than 35 kcal/mol. A third pathway, in which the ICl_3_ catalyst forms covalent bonds with l-lactide instead of through noncovalent XB, was calculated to be more favorable compared to XB-catalysis pathways.

The apparent high barriers of the XB pathways, notwithstanding the exclusion of the entropic component which could further add to the reaction barriers, indicate that other mechanistic pathways other than the reported four-membered ring TSs might be in operation. Furthermore, as noted above, hypervalent iodine(III) compounds are often used as reagents instead of catalysts. In the presence of an alcohol reagent **54**, the possibility of Brønsted acid generation should be thoroughly investigated and compared to the XB catalysis pathway.

## 4. Summary and Outlook

Halogen bonding is now established clearly as a reliable noncovalent motif in organocatalysis. We witnessed a continued progress in the application of XB to organocatalysis in recent years. Although computational mechanistic studies of reactions catalyzed by halogen-containing catalysts are still very few, there seems to now be sufficient computational evidence in support of the possibility of XB activation in organocatalysis. Calculated TS structures reported in the literature showed that the catalysts employed can activate substrates through interaction at the halogen site, with features characteristic of a typical halogen bond, e.g., close to linear binding angle and less than the sum of van der Waals radii binding distance. Various energy decomposition analyses of the strength of XB binding seem to indicate that it could be strong enough to attain a lowering of the reaction free energy barrier.

To provide stronger support for the XB activation mode through computational studies, several practices are recommended in future studies. Firstly, the entropic contribution to reaction barrier, if possible, should be included, or at least assessed in some empirical ways when it is difficult to calculate. Failing to include it may result in overestimation of XB activation with respect to the uncatalyzed reaction and poor agreement with experiments. Secondly, for cationic halogen bond catalysts, the effect of counter anions on the reaction barriers needs to be examined. It is common practice to omit them when calculating catalyzed transition states, assuming they have a minimal effect. Experimentally, there is evidence that the effect may not be as minimal as once thought. On the other hand, calculated reaction free energy barriers are sometimes rather close to those of uncatalyzed reactions. Thirdly, comparison to other possible pathways is highly desirable, especially to the Brønsted acid pathway. It was noted experimentally and theoretically by Breugst et al. [81], Scheidt et al. [28], and our group [43,92] that the Brønsted acid catalysis pathway is competitive or superior to the XB catalysis pathway for the investigated reactions. However, the understanding of how a trace amount of acid could be generated is quite limited, perhaps because there are not many experimental reports, if any, of such a nature. Further computational studies founded on more extensive evidence are needed. Finally, a whole reaction profile, detailing every elementary step, is necessary to understand the reaction kinetics, to which, corresponding experimental data, e.g., reaction orders and kinetic isotope effects, could be directly compared (if they exist).

Computational studies could also help to advance this growing field through screening new catalysts before experimental synthesis of selected top candidates. The experimental design of new and novel XB catalysts is always highly sought after but very challenging, owing to the fact that, to overcome the unfavorable entropy penalty, it is usually necessary to form multiple halogen bonding interactions with the substrates. Furthermore, XB is highly directional and usually weaker than the corresponding hydrogen bond, and design of multidentate XB catalysts to achieve close-to-optimal binding in key transition states poses a very strict requirement on molecular geometry. Moreover, the atom σ-bonded to the halogen atoms is currently restricted only to carbon, while other atoms like nitrogen and oxygen, which are commonly found in hydrogen bond catalysis, will render the σ bond liable to homolysis or other reaction channels.

On the other hand, the computational design of new halogen bond catalysts can benefit from the speed of computational design relative to actual synthesis and experimental testing of designed catalysts, which is both challenging and tedious. More importantly, it seems quite plausible that computational design could also benefit from design strategies already well developed in the field of de novo drug design. The quest for a scaffold or linker connecting isolated halogen bond donors to achieve maximal catalyst–substrate interaction parallels well to computer-aided drug discovery. Reports of such an approach might be expected in the near future. Thus, we believe that in silico design will play a very important role in designing the right catalyst with optimal halogen bonding interactions with the substrate.

Lastly, asymmetric catalysis utilizing halogen bond as a control element is starting to appear in literature [95]. We expect that many more papers may appear, and the strategy may mature to a standard protocol in organocatalysis. We certainly will see more contribution by the computational chemistry community in this respect. In summary, theoretical studies will continue to play critical roles in the mechanistic understanding and design of next-generation novel XB-based organocatalysts.

## Figures and Tables

**Table 1:** Calculated binding energies (ΔG_298_ and ΔG_298_) and interaction distances (D_XB_) of the XB complex between iodopentafluorobenzene and triethylamine using various density functional theory (DFT) methods.

DFT ^a^	ΔH_298_ (kcal/mol)	ΔG_298_^b^ (kcal/mol)	D_XB_ (Å)
MN15	−10.1	−1.0	2.75
B3LYP	−1.6	7.2	2.87
B3LYP-D3	−8.1	1.4	2.79
M06-2X	−8.2	1.2	2.80
ω-B97XD	−6.9	2.4	2.85
PBE0	−4.1	5.0	2.78
PBE0-D3	−8.2	1.1	2.74

^a^ All calculations were performed at the DFT/6-311++G(2d,p)//DFT/6-31+G(d) level. Detailed computational results are given in Supplementary Materials. Free energy corrections were calculated at 298 K and with correction to the standard state. ^b^ Experimentally determined binding free energy at 298 K in cyclohexane: ΔG_298_ = −0.2 ± 0.1 kcal/mol [51].

**Table 2:**
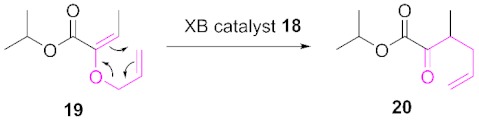
Comparison of reaction kinetics between uncatalyzed and CAT-3-perFPhI-catalyzed Claisen rearrangement reaction.

	Uncatalyzed		18-Catalyzed	
T(k)	ΔG^≠^ (kcal/mol)	kK_eq_ (M h^−1^)	ΔG^≠^*_n_*_-hexane_ (kcal/mol)	TOF (h^−1^)
318.15	26.5	5.3×10^−4^	28.3	8.6×10^−4^
298.15	26.5	3.3×10^−5^	27.3	2.3×10^−4^
273.15	26.4	5.3×10^−7^	26.0	2.5×10^−5^

## Supplementary Materials

The following are available online, Table S1: Cartesian coordinates (Å), thermodynamics of the optimized structures at DFT/6-31G+(d)+def2-SVP(I) level, and single point energies at DFT/6-311++G(2d,p)+def2-TZVPD(I)level.
