# Supplementary material for: Application of Halogen Bonding to Organocatalysis: A Theoretical Perspective
Source: Molecules. 2020 Feb 26;25(5):1045. doi: 10.3390/molecules25051045 (PMC7179134; doi:10.3390/molecules25051045)
Supplement: Supplementary file 1 [file molecules-25-01045-s001.pdf]

molecules

Review

Application of Halogen Bonding to Organocatalysis: A Theoretical Perspective

Hui Yang and Ming Wah Wong *

Department of Chemistry, National University of Singapore, 3 Science Drive 3, Singapore 117543; chmwmw@nus.edu.sg
* Correspondence: chmwmw@nus.edu.sg; Tel.: +65-6516-4320; Fax: +65-6779-1691.

Academic Editor: Paulo Jorge Costa

Table of Contents

Computational Details 1
Table S1 1
References 15

Computational Details

All geometries reported were optimized at SMD(cyclohexane)/DFT/6-311++G(2d,p)//SMD(cyclohexane)/DFT/6-31+G(d) level. DFT methods investigated included B3LYP, B3LYP-D3, MN15, M06-2X, ω-B97XD, PBE0, and PBE0-D3. For iodine atoms, the def2-SVP basis set was for optimization and the def2-TZVPD basis set was used for single point energy calculation. The refined SMD18 radius for iodine was employed in all calculations. Free energy corrections were calculated at 298 K and with correction to standard state. All calculations were carried out the Gaussian 16 suite of programs [1].

Table S1.

Table S1. Cartesian coordinates (Å), thermodynamics of the optimized structures at DFT/6-31G+(d)+def2-SVP(I) level, and single point energies at DFT/6-311+G(2d,p)+def2-TZVPD(I) level.

B3LYPD3-COMP-C-HEXANE

C	4.26435200	0.00585200	-0.00466500
C	1.44570300	-0.00379400	-0.00059200
C	3.56896000	-1.19837300	0.06818100
C	3.56062000	1.20534800	-0.07542200
C	2.16767600	1.18606300	-0.07242900
C	2.17586100	-1.18869100	0.06948700
F	5.60580500	0.01039300	-0.00669200
F	4.23049600	2.36883300	-0.14517000
F	1.53029900	2.37296900	-0.14203800
F	1.54683900	-2.37986900	0.14068400
F	4.24698000	-2.35724500	0.13606200

I	-0.71775900	-0.00875300	0.00280100
N	-3.50542400	0.00002400	0.00724700
C	-3.95511800	0.51844500	1.32121100
H	-5.04859100	0.68565900	1.29502100
H	-3.48176400	1.49322200	1.46323000
C	-3.96993100	-1.39221600	-0.20162800
H	-5.06427900	-1.44083600	-0.04545300
H	-3.50453200	-2.00757500	0.57240200
C	-3.95742300	0.88254600	-1.09494200
H	-5.05241300	0.78271400	-1.21887100
H	-3.49277600	0.51591000	-2.01376300
C	-3.62446100	-1.99413000	-1.56451700
H	-3.84602600	-3.06766900	-1.54127900
H	-2.55988200	-1.87801500	-1.79590300
H	-4.20762100	-1.55854400	-2.38221500
C	-3.59792800	2.36033800	-0.93247000
H	-4.17822800	2.85528700	-0.14717800
H	-3.81292500	2.88002200	-1.87370300
H	-2.53270900	2.49282000	-0.71323100
C	-3.60579600	-0.36407500	2.52060900
H	-2.54299100	-0.63017300	2.52644400
H	-4.19444700	-1.28634800	2.55578900
H	-3.81658900	0.19323800	3.44101500

Zero-point correction= 0.258060 (Hartree/Particle)
Thermal correction to Energy= 0.279336
Thermal correction to Enthalpy= 0.280280
Thermal correction to Gibbs Free Energy= 0.205332
Sum of electronic and zero-point Energies= -1317.818586
Sum of electronic and thermal Energies= -1317.797310
Sum of electronic and thermal Enthalpies= -1317.796366
Sum of electronic and thermal Free Energies= -1317.871315
E(RB3LYP/6-311+G(2d,p)+def2-TZVPD(I)) = -1318.38409494

B3LYPD3-F5PhI-C-HEXANE

C	-0.51358200	-0.70842600	0.74734500
C	-0.25690700	-0.59897800	3.54208700
C	0.36860700	-1.56482600	1.40141600
C	-1.26752500	0.20288700	1.48264900
C	-1.13571400	0.25163500	2.86866000
C	0.49066600	-1.50550100	2.78792900
F	-0.63717400	-0.76148600	-0.58417000
F	-2.11744600	1.02764600	0.85367700
F	-1.88022600	1.14348800	3.54040700
F	1.35186800	-2.34713300	3.38032700
F	1.09524900	-2.44268000	0.69465100
I	-0.06343600	-0.51644300	5.64019500

Zero-point correction= 0.049105 (Hartree/Particle)
Thermal correction to Energy= 0.059479
Thermal correction to Enthalpy= 0.060423
Thermal correction to Gibbs Free Energy= 0.011324
Sum of electronic and zero-point Energies= -1025.565692
Sum of electronic and thermal Energies= -1025.555318

Sum of electronic and thermal Enthalpies= -1025.554374
Sum of electronic and thermal Free Energies= -1025.603473
E(RB3LYP/6-311+G(2d,p)+def2-TZVPD(I)) = -1025.83895806

B3LYPD3-NEt3-C-HEXANE

N	0.00092400	-0.00014300	0.06835100
C	0.51325100	1.30891000	0.49846100
H	0.83509900	1.27457500	1.55914600
H	-0.31001300	2.02869600	0.45179200
C	0.87831000	-1.09887000	0.49726000
H	0.69111500	-1.35807400	1.55916900
H	1.91373400	-0.74775300	0.44604500
C	-1.38890400	-0.21105900	0.49798900
H	-1.51978300	0.08211800	1.55948000
H	-1.60130500	-1.28357300	0.44832700
C	0.75164300	-2.34524400	-0.38036800
H	1.44379600	-3.12417000	-0.03537900
H	0.99154300	-2.10157100	-1.42194800
H	-0.25897100	-2.76924100	-0.35796700
C	-2.40566200	0.51952300	-0.38065300
H	-2.26682700	1.60666200	-0.36154900
H	-3.42607500	0.31114000	-0.03402300
H	-2.31584300	0.18686400	-1.42134700
C	1.65179000	1.82663600	-0.38212700
H	1.31592700	1.91754500	-1.42172400
H	2.52422000	1.16314300	-0.36710600
H	1.98203200	2.81388800	-0.03410300

Zero-point correction= 0.206466 (Hartree/Particle)
Thermal correction to Energy= 0.215810
Thermal correction to Enthalpy= 0.216754
Thermal correction to Gibbs Free Energy= 0.172695
Sum of electronic and zero-point Energies= -292.241163
Sum of electronic and thermal Energies= -292.231819
Sum of electronic and thermal Enthalpies= -292.230875
Sum of electronic and thermal Free Energies= -292.274934
E(RB3LYP/6-311+G(2d,p)+def2-TZVPD(I)) = -292.529078939

B3LYP-COMP-C-HEXANE

C	4.30871400	0.00196500	-0.00127800
C	1.49198600	-0.00075500	-0.00131800
C	3.61059400	-1.20049200	-0.07187700
C	3.60830500	1.20309600	0.06921800
C	2.21551800	1.18774800	0.06855400
C	2.21776300	-1.18790200	-0.07090300
F	5.64971300	0.00324500	-0.00131600
F	4.28044500	2.36476000	0.13666200
F	1.57976000	2.37421600	0.13740800
F	1.58434500	-2.37556300	-0.14004600
F	4.28498100	-2.36084800	-0.13944600
I	-0.66113800	-0.00228700	0.00029000
N	-3.53240700	0.00108100	0.00576100
C	-3.99182900	0.53865600	1.30906800

H	-5.09132400	0.66623900	1.28550800
H	-3.55663700	1.53492400	1.42078500
C	-4.00436600	-1.39246100	-0.17917300
H	-5.10391100	-1.42571800	-0.05409100
H	-3.57263400	-1.99085700	0.62697300
C	-3.99890100	0.86223800	-1.10769500
H	-5.09898000	0.77757400	-1.19947000
H	-3.56940100	0.46163600	-2.02947100
C	-3.63071800	-2.04365500	-1.51213100
H	-3.87766400	-3.11096700	-1.46153800
H	-2.55733900	-1.96079000	-1.71606400
H	-4.17884700	-1.62479200	-2.36221700
C	-3.61636300	2.33981000	-1.00255400
H	-4.16282300	2.86834000	-0.21478800
H	-3.85868500	2.83271700	-1.95180500
H	-2.54269100	2.46846800	-0.82557500
C	-3.60932100	-0.29192900	2.53570100
H	-2.53643200	-0.51379400	2.55521200
H	-4.15944600	-1.23606900	2.60221000
H	-3.84628900	0.28554600	3.43742800

Zero-point correction= 0.257211 (Hartree/Particle)
Thermal correction to Energy= 0.278841
Thermal correction to Enthalpy= 0.279785
Thermal correction to Gibbs Free Energy= 0.203446
Sum of electronic and zero-point Energies= -1317.782408
Sum of electronic and thermal Energies= -1317.760778
Sum of electronic and thermal Enthalpies= -1317.759834
Sum of electronic and thermal Free Energies= -1317.836174
E(RB3LYP/6-311+G(2d,p)+def2-TZVPD(I)) = -1318.34741563

B3LYP-F5PhI-C-HEXANE

C	-0.51355000	-0.70841300	0.74768500
C	-0.25701900	-0.59902300	3.54081500
C	0.36856700	-1.56477800	1.40144800
C	-1.26747400	0.20284400	1.48267600
C	-1.13535600	0.25123900	2.86845300
C	0.49028200	-1.50511000	2.78775800
F	-0.63714400	-0.76147400	-0.58370200
F	-2.11731100	1.02751500	0.85382500
F	-1.87856600	1.14199700	3.54245400
F	1.35060900	-2.34546100	3.38252300
F	1.09513900	-2.44254300	0.69481200
I	-0.06379800	-0.51661000	5.63642300

Zero-point correction= 0.049145 (Hartree/Particle)
Thermal correction to Energy= 0.059525
Thermal correction to Enthalpy= 0.060469
Thermal correction to Gibbs Free Energy= 0.011352
Sum of electronic and zero-point Energies= -1025.555454
Sum of electronic and thermal Energies= -1025.545074
Sum of electronic and thermal Enthalpies= -1025.544130
Sum of electronic and thermal Free Energies= -1025.593247
E(RB3LYP/6-311+G(2d,p)+def2-TZVPD(I)) = -1025.82883360

B3LYP-NEt3-C-HEXANE

N	0.00075900	-0.00009000	0.06075400
C	0.51340500	1.30912100	0.49185800
H	0.82688800	1.27660700	1.55513100
H	-0.30987000	2.02829100	0.43720200
C	0.87789200	-1.09925300	0.49090800
H	0.69567500	-1.35273000	1.55511000
H	1.91267600	-0.74736700	0.43276300
C	-1.38987800	-0.21055500	0.48999900
H	-1.51945600	0.07532600	1.55367600
H	-1.60157600	-1.28282000	0.43302100
C	0.75100000	-2.35398800	-0.37509000
H	1.44886000	-3.12546200	-0.02479400
H	0.98618700	-2.12307700	-1.42065600
H	-0.25634300	-2.78507400	-0.34352300
C	-2.41259400	0.52485100	-0.37806700
H	-2.28090000	1.61268000	-0.35003700
H	-3.42985600	0.30851200	-0.02697600
H	-2.33074700	0.20233000	-1.42262400
C	1.66039100	1.82977200	-0.37632500
H	1.33865100	1.92332500	-1.42015900
H	2.53669800	1.17170700	-0.35169600
H	1.98213900	2.81789100	-0.02274600

Zero-point correction= 0.206198 (Hartree/Particle)
Thermal correction to Energy= 0.215607
Thermal correction to Enthalpy= 0.216551
Thermal correction to Gibbs Free Energy= 0.172361
Sum of electronic and zero-point Energies= -292.225633
Sum of electronic and thermal Energies= -292.216225
Sum of electronic and thermal Enthalpies= -292.215281
Sum of electronic and thermal Free Energies= -292.259471
E(RB3LYP/6-311+G(2d,p)+def2-TZVPD(I)) = -292.513269652

M062X-COMP-C-HEXANE

C	4.23922600	0.00710500	-0.00566400
C	1.43250000	-0.00345600	-0.00009000
C	3.54742000	-1.19396500	0.06382100
C	3.53822200	1.20300200	-0.07225800
C	2.14980800	1.18382800	-0.06864800
C	2.15885100	-1.18530800	0.06590000
F	5.56976900	0.01207700	-0.00843800
F	4.20140000	2.35753400	-0.13799500
F	1.51486700	2.35817700	-0.13347900
F	1.53317200	-2.36438800	0.13304300
F	4.21948600	-2.34348700	0.12701900
I	-0.68906900	-0.00922100	0.00360900
N	-3.48454100	-0.00089500	0.00764100
C	-3.93735000	0.52620400	1.30496500
H	-5.02931600	0.70411500	1.27114000
H	-3.45504000	1.49673500	1.45166000
C	-3.95359400	-1.38230700	-0.18643800

H	-5.04686100	-1.42806500	-0.01946300
H	-3.48181600	-2.00048100	0.58250300
C	-3.93752300	0.86389000	-1.09380100
H	-5.03121200	0.75400700	-1.22400300
H	-3.46389800	0.50270700	-2.01108700
C	-3.62378200	-1.97576600	-1.55116200
H	-3.83335800	-3.04966400	-1.53070800
H	-2.56396700	-1.84680300	-1.79582200
H	-4.22196800	-1.54025100	-2.35606200
C	-3.59309000	2.33893000	-0.92224400
H	-4.19088300	2.82373500	-0.14576600
H	-3.79214300	2.86099500	-1.86323800
H	-2.53334600	2.47567600	-0.68146700
C	-3.60556500	-0.36272200	2.49804600
H	-2.54874600	-0.65059500	2.49808200
H	-4.21273000	-1.27140700	2.52835400
H	-3.80094600	0.19344500	3.42007300

Zero-point correction= 0.260873 (Hartree/Particle)
Thermal correction to Energy= 0.282012
Thermal correction to Enthalpy= 0.282956
Thermal correction to Gibbs Free Energy= 0.208328
Sum of electronic and zero-point Energies= -1317.207199
Sum of electronic and thermal Energies= -1317.186061
Sum of electronic and thermal Enthalpies= -1317.185117
Sum of electronic and thermal Free Energies= -1317.259744
E(RM062X/6-311+G(2d,p)+def2-TZVPD(I)) = -1317.78813837

M062X-F5PhI-C-HEXANE

C	-0.51288600	-0.70810200	0.75376400
C	-0.25771900	-0.59938400	3.53606700
C	0.36641400	-1.56238400	1.40474700
C	-1.26491300	0.20048400	1.48570700
C	-1.13320300	0.24831400	2.86705500
C	0.48724300	-1.50264100	2.78661100
F	-0.63522100	-0.76037400	-0.56751600
F	-2.10791100	1.01880000	0.86125600
F	-1.87039200	1.13142000	3.53746800
F	1.34048300	-2.33586800	3.37854600
F	1.08758600	-2.43303700	0.70322200
I	-0.06510200	-0.51704500	5.60824500

Zero-point correction= 0.050539 (Hartree/Particle)
Thermal correction to Energy= 0.060795
Thermal correction to Enthalpy= 0.061739
Thermal correction to Gibbs Free Energy= 0.012901
Sum of electronic and zero-point Energies= -1025.134256
Sum of electronic and thermal Energies= -1025.123999
Sum of electronic and thermal Enthalpies= -1025.123055
Sum of electronic and thermal Free Energies= -1025.171894
E(RM062X/6-311+G(2d,p)+def2-TZVPD(I)) = -1025.41960111

M062x-NEt3-C-HEXANE

N	-0.00029400	0.00007400	-0.14755600

C	0.39137200	1.33608600	0.30128700
H	0.37360100	1.39442300	1.40954100
H	-0.35803800	2.04124000	-0.06683100
C	0.96092100	-1.00687300	0.30199100
H	1.01936800	-1.02053400	1.41030900
H	1.94638200	-0.70961000	-0.06514600
C	-1.35305200	-0.32898200	0.30149400
H	-1.39435500	-0.37417800	1.40974500
H	-1.58865300	-1.33041800	-0.06702700
C	0.67160200	-2.41244300	-0.21503900
H	1.54504400	-3.05082900	-0.04742900
H	0.46849500	-2.38657600	-1.29132200
H	-0.17676900	-2.88558900	0.28712000
C	-2.42503300	0.62554400	-0.21450200
H	-2.41150400	1.59507000	0.29092900
H	-3.41483700	0.18761000	-0.05011000
H	-2.29902600	0.79195100	-1.29000000
C	1.75381200	1.78673200	-0.21573800
H	1.83316300	1.59606600	-1.29166200
H	2.58713300	1.28870300	0.28751000
H	1.87066600	2.86253400	-0.04983400

Zero-point correction= 0.208629 (Hartree/Particle)
Thermal correction to Energy= 0.217847
Thermal correction to Enthalpy= 0.218791
Thermal correction to Gibbs Free Energy= 0.174985
Sum of electronic and zero-point Energies= -292.059673
Sum of electronic and thermal Energies= -292.050455
Sum of electronic and thermal Enthalpies= -292.049511
Sum of electronic and thermal Free Energies= -292.093317
E(RM062X/6-311+G(2d,p)+def2-TZVPD(I)) = -292.353071692

MN15-COMP-C-HEXANE

C	-0.45178500	-0.74539100	0.84387900
C	-0.28783600	-0.55692300	3.64600800
C	-0.01988900	-1.83532400	1.59268200
C	-0.80312800	0.43791900	1.48545200
C	-0.71711800	0.51675300	2.87210600
C	0.05625500	-1.72803600	2.97808400
F	-0.52954500	-0.83491100	-0.48183800
F	-1.21835100	1.48292200	0.76906600
F	-1.06398400	1.67348700	3.44831000
F	0.47706500	-2.79994800	3.65935900
F	0.31657300	-2.97010400	0.97893300
I	-0.15871400	-0.40912200	5.76850700
N	0.00930300	-0.20646800	8.50727800
C	1.05533700	0.78011200	8.82580900
H	1.00944200	1.02356000	9.90726600
H	0.82091000	1.69863300	8.27488200
C	0.35712500	-1.53180000	9.04746000
H	0.61666500	-1.43060900	10.12140200
H	1.25793800	-1.87489600	8.52514400
C	-1.30069200	0.23773900	9.01326000

H	-1.31583300	0.14193000	10.11848700
H	-2.05763600	-0.44909200	8.61624400
C	-0.73169700	-2.58623100	8.88227000
H	-0.31729200	-3.57051300	9.12908100
H	-1.09201500	-2.62412800	7.84578600
H	-1.58881400	-2.41433700	9.54276500
C	-1.68168700	1.66065600	8.61937700
H	-1.09193500	2.41732800	9.14870200
H	-2.73546200	1.83069900	8.86875900
H	-1.56231200	1.81918500	7.53955200
C	2.46927400	0.34178800	8.46067200
H	2.51666900	-0.01982900	7.42503200
H	2.85096600	-0.44496100	9.12074600
H	3.14421100	1.20119300	8.54721500

Zero-point correction= 0.258968 (Hartree/Particle)
Thermal correction to Energy= 0.280218
Thermal correction to Enthalpy= 0.281162
Thermal correction to Gibbs Free Energy= 0.205859
Sum of electronic and zero-point Energies= -1315.901662
Sum of electronic and thermal Energies= -1315.880412
Sum of electronic and thermal Enthalpies= -1315.879468
Sum of electronic and thermal Free Energies= -1315.954771
E(RMN15/6-311+G(2d,p)+def2-TZVPD(I)) = -1316.49099655

MN15-F5PhI-C-HEXANE

C	-0.51325800	-0.70837100	0.75324500
C	-0.25738600	-0.59917900	3.53266100
C	0.36583500	-1.56198400	1.40455400
C	-1.26501000	0.19959700	1.48544100
C	-1.13913500	0.25575500	2.87047400
C	0.49493600	-1.50931300	2.78943500
F	-0.63600100	-0.76077300	-0.58018900
F	-2.11448000	1.02462100	0.85769000
F	-1.87861800	1.14349600	3.55206500
F	1.35250000	-2.34662600	3.39185700
F	1.09250600	-2.43944200	0.69861800
I	-0.06751100	-0.51760000	5.59932200

Zero-point correction= 0.050231 (Hartree/Particle)
Thermal correction to Energy= 0.060568
Thermal correction to Enthalpy= 0.061512
Thermal correction to Gibbs Free Energy= 0.012478
Sum of electronic and zero-point Energies= -1024.069189
Sum of electronic and thermal Energies= -1024.058852
Sum of electronic and thermal Enthalpies= -1024.057908
Sum of electronic and thermal Free Energies= -1024.106942
E(RMN15/6-311+G(2d,p)+def2-TZVPD(I)) = -1024.36109700

MN15-NEt3-C-HEXANE

N	0.00000000	0.00000000	-0.30302000
C	0.36738200	1.35586600	0.18817400
H	0.22152300	1.42116200	1.27438700
H	-0.30126900	2.09100000	-0.27409500

C	0.99052400	-0.99609500	0.18817400
H	1.12000100	-0.90242600	1.27438700
H	1.96149400	-0.78459300	-0.27409500
C	-1.35790500	-0.35977100	0.18817400
H	-1.34152400	-0.51873700	1.27438700
H	-1.66022500	-1.30640700	-0.27409500
C	0.68173000	-2.45842100	-0.13281500
H	1.54027700	-3.08348400	0.13581100
H	0.48561000	-2.60710900	-1.19827100
H	-0.17608900	-2.82802000	0.43705900
C	-2.46992000	0.63881500	-0.13281500
H	-2.36109300	1.56650700	0.43705900
H	-3.44051400	0.20782300	0.13581100
H	-2.50062800	0.88300400	-1.19827100
C	1.78819000	1.81960600	-0.13281500
H	2.01501800	1.72410500	-1.19827100
H	2.53718200	1.26151300	0.43705900
H	1.90023700	2.87566100	0.13581100

Zero-point correction= 0.207176 (Hartree/Particle)
Thermal correction to Energy= 0.216453
Thermal correction to Enthalpy= 0.217397
Thermal correction to Gibbs Free Energy= 0.173525
Sum of electronic and zero-point Energies= -291.831301
Sum of electronic and thermal Energies= -291.822024
Sum of electronic and thermal Enthalpies= -291.821080
Sum of electronic and thermal Free Energies= -291.864953
E(RMN15/6-311+G(2d,p)+def2-TZVPD(I)) = -292.111502730

PBE0D3-COMP-C-HEXANE

C	4.23093400	0.00218900	0.00498400
C	1.41773700	-0.00201100	-0.00795300
C	3.53470900	-1.20222200	0.00120200
C	3.53125900	1.20456500	0.00142400
C	2.13983200	1.18738800	-0.00512700
C	2.14318800	-1.18935900	-0.00523900
F	5.56190300	0.00413500	0.01106400
F	4.19718300	2.35962400	0.00423700
F	1.50678000	2.36623100	-0.00818700
F	1.51390400	-2.37017900	-0.00814300
F	4.20414900	-2.35523400	0.00412600
I	-0.71382200	-0.00327400	-0.00709300
N	-3.46514600	0.00137900	0.00511100
C	-3.91266400	0.55095900	1.29626300
H	-5.00417800	0.74449100	1.25117400
H	-3.41561500	1.51866900	1.43274100
C	-3.92801800	-1.38695400	-0.16157400
H	-5.01932500	-1.43277100	0.03324500
H	-3.43384300	-1.99375700	0.60627700
C	-3.92531000	0.84504600	-1.11106600
H	-5.01823800	0.70853000	-1.24452400
H	-3.43768800	0.48036900	-2.02293900
C	-3.62720600	-1.99367600	-1.52757700

H -3.82880000 -3.07057600 -1.49293700
H -2.57146900 -1.85851600 -1.79689800
H -4.24720500 -1.56757900 -2.32411000
C -3.61203900 2.32895500 -0.95275000
H -4.22783100 2.81052400 -0.18501600
H -3.81026200 2.83975300 -1.90218700
H -2.55513600 2.48675400 -0.70081000
C -3.59956800 -0.33048300 2.50048000
H -2.54557300 -0.63807500 2.50246400
H -4.22375500 -1.23013900 2.53852900
H -3.78490900 0.23862300 3.41883100
Zero-point correction= 0.260117 (Hartree/Particle)
Thermal correction to Energy= 0.281302
Thermal correction to Enthalpy= 0.282246
Thermal correction to Gibbs Free Energy= 0.207102
Sum of electronic and zero-point Energies= -1316.639970
Sum of electronic and thermal Energies= -1316.618785
Sum of electronic and thermal Enthalpies= -1316.617841
Sum of electronic and thermal Free Energies= -1316.692985
E(RPBE1PBE/6-311+G(2d,p)+def2-TZVPD(I)) = -1317.18996736

PBE0D3-F5PhI-C-HEXANE
C -0.51325800 -0.70837100 0.75324500
C -0.25738600 -0.59917900 3.53266100
C 0.36583500 -1.56198400 1.40455400
C -1.26501000 0.19959700 1.48544100
C -1.13913500 0.25575500 2.87047400
C 0.49493600 -1.50931300 2.78943500
F -0.63600100 -0.76077300 -0.58018900
F -2.11448000 1.02462100 0.85769000
F -1.87861800 1.14349600 3.55206500
F 1.35250000 -2.34662600 3.39185700
F 1.09250600 -2.43944200 0.69861800
I -0.06751100 -0.51760000 5.59932200
Zero-point correction= 0.050170 (Hartree/Particle)
Thermal correction to Energy= 0.060457
Thermal correction to Enthalpy= 0.061401
Thermal correction to Gibbs Free Energy= 0.012488
Sum of electronic and zero-point Energies= -1024.760449
Sum of electronic and thermal Energies= -1024.750162
Sum of electronic and thermal Enthalpies= -1024.749218
Sum of electronic and thermal Free Energies= -1024.798131
E(RPBE1PBE/6-311+G(2d,p)+def2-TZVPD(I)) = -1025.02443503

PBE0D3-NEt3-C-HEXANE
N 0.00000000 0.00000000 -0.30302000
C 0.36738200 1.35586600 0.18817400
H 0.22152300 1.42116200 1.27438700
H -0.30126900 2.09100000 -0.27409500
C 0.99052400 -0.99609500 0.18817400
H 1.12000100 -0.90242600 1.27438700
H 1.96149400 -0.78459300 -0.27409500

C	-1.35790500	-0.35977100	0.18817400
H	-1.34152400	-0.51873700	1.27438700
H	-1.66022500	-1.30640700	-0.27409500
C	0.68173000	-2.45842100	-0.13281500
H	1.54027700	-3.08348400	0.13581100
H	0.48561000	-2.60710900	-1.19827100
H	-0.17608900	-2.82802000	0.43705900
C	-2.46992000	0.63881500	-0.13281500
H	-2.36109300	1.56650700	0.43705900
H	-3.44051400	0.20782300	0.13581100
H	-2.50062800	0.88300400	-1.19827100
C	1.78819000	1.81960600	-0.13281500
H	2.01501800	1.72410500	-1.19827100
H	2.53718200	1.26151300	0.43705900
H	1.90023700	2.87566100	0.13581100

Zero-point correction= 0.207746 (Hartree/Particle)
Thermal correction to Energy= 0.217072
Thermal correction to Enthalpy= 0.218016
Thermal correction to Gibbs Free Energy= 0.174001
Sum of electronic and zero-point Energies= -291.866145
Sum of electronic and thermal Energies= -291.856819
Sum of electronic and thermal Enthalpies= -291.855874
Sum of electronic and thermal Free Energies= -291.899890
E(RPBE1PBE/6-311+G(2d,p)+def2-TZVPD(I)) = -292.149644306

PBE0-COMP-C-HEXANE

C	4.23150700	0.00520500	-0.00389100
C	1.42438900	-0.00296100	-0.00025600
C	3.53834100	-1.19558000	0.07706400
C	3.53124300	1.20198900	-0.08297700
C	2.14206800	1.18414500	-0.08017900
C	2.14903400	-1.18590100	0.07802400
F	5.56236400	0.00903600	-0.00568900
F	4.19670700	2.35508900	-0.16026600
F	1.50760000	2.35862300	-0.15746700
F	1.52164000	-2.36399200	0.15680000
F	4.21070900	-2.34478300	0.15268000
I	-0.70840900	-0.00710300	0.00261500
N	-3.48477000	0.00013500	0.00698600
C	-3.93952400	0.50805000	1.31019600
H	-5.03553800	0.66322400	1.28146700
H	-3.48102100	1.49074800	1.45498000
C	-3.95421100	-1.37749100	-0.20517100
H	-5.05085700	-1.41794200	-0.05711800
H	-3.50304000	-1.99902100	0.57387700
C	-3.94307200	0.87788100	-1.08064800
H	-5.04049300	0.78081700	-1.19225500
H	-3.49354700	0.50977900	-2.00766500
C	-3.60746800	-1.97816400	-1.55884000
H	-3.84092400	-3.04883300	-1.53900100
H	-2.53977200	-1.87682200	-1.78404800
H	-4.17936200	-1.53776400	-2.38176200

C	-3.58241500	2.34708900	-0.92266600
H	-4.15078000	2.84415200	-0.13017500
H	-3.81025900	2.86806100	-1.85967400
H	-2.51383100	2.48175000	-0.72031800
C	-3.58737300	-0.36671500	2.50369400
H	-2.52106200	-0.61921900	2.51837100
H	-4.16417000	-1.29637000	2.53782700
H	-3.80972600	0.18627000	3.42350700

Zero-point correction= 0.259753 (Hartree/Particle)
Thermal correction to Energy= 0.281090
Thermal correction to Enthalpy= 0.282034
Thermal correction to Gibbs Free Energy= 0.206466
Sum of electronic and zero-point Energies= -1316.617728
Sum of electronic and thermal Energies= -1316.596391
Sum of electronic and thermal Enthalpies= -1316.595447
Sum of electronic and thermal Free Energies= -1316.671015
E(RPBE1PBE/6-311+G(2d,p)+def2-TZVPD(I)) = -1317.16749870

PBE0-F5PhI-C-HEXANE

C	-0.51306200	-0.70822800	0.75300300
C	-0.25738300	-0.59914100	3.53614800
C	0.36718600	-1.56298800	1.40434400
C	-1.26567100	0.20114400	1.48540500
C	-1.13413100	0.24986200	2.86753600
C	0.48901700	-1.50370500	2.78697500
F	-0.63564600	-0.76073200	-0.56816900
F	-2.10877800	1.01921300	0.86085900
F	-1.87051800	1.13285400	3.53819900
F	1.34195700	-2.33657900	3.37906000
F	1.08783900	-2.43380900	0.70260700
I	-0.06643100	-0.51771100	5.60920600

Zero-point correction= 0.050192 (Hartree/Particle)
Thermal correction to Energy= 0.060483
Thermal correction to Enthalpy= 0.061427
Thermal correction to Gibbs Free Energy= 0.012503
Sum of electronic and zero-point Energies= -1024.754371
Sum of electronic and thermal Energies= -1024.744080
Sum of electronic and thermal Enthalpies= -1024.743136
Sum of electronic and thermal Free Energies= -1024.792060
E(RPBE1PBE/6-311+G(2d,p)+def2-TZVPD(I)) = -1025.01842144

PBE0-NEt3-C-HEXANE

N	0.00089200	0.00023200	0.05024600
C	0.50479900	1.29960600	0.48471700
H	0.80742600	1.26657700	1.55126100
H	-0.31843100	2.01917800	0.42398600
C	0.87444900	-1.08652100	0.48235700
H	0.69910000	-1.33022000	1.55013000
H	1.90948100	-0.73515100	0.41611600
C	-1.37669500	-0.21322700	0.48325000
H	-1.50040300	0.06437800	1.54993300
H	-1.58837800	-1.28576500	0.42114900

C	0.74256700	-2.34007000	-0.36946300
H	1.44314900	-3.10929700	-0.02219500
H	0.96804700	-2.11429000	-1.41773500
H	-0.26348400	-2.77245200	-0.32755800
C	-2.39706300	0.52314700	-0.37167700
H	-2.26751800	1.61068900	-0.33658300
H	-3.41335100	0.30387000	-0.02209500
H	-2.31552700	0.20866900	-1.41816400
C	1.65225800	1.81708000	-0.36963600
H	1.33901800	1.90508000	-1.41599100
H	2.52995500	1.16187500	-0.33567800
H	1.96971100	2.80661200	-0.01864400

Zero-point correction= 0.207562 (Hartree/Particle)
Thermal correction to Energy= 0.216944
Thermal correction to Enthalpy= 0.217889
Thermal correction to Gibbs Free Energy= 0.173720
Sum of electronic and zero-point Energies= -291.856579
Sum of electronic and thermal Energies= -291.847197
Sum of electronic and thermal Enthalpies= -291.846253
Sum of electronic and thermal Free Energies= -291.890422
E(RPBE1PBE/6-311+G(2d,p)+def2-TZVPD(I)) = -292.139895993

wb97xd-COMP-C-HEXANE

C	4.27673800	0.00478400	-0.00768300
C	1.46736700	-0.00252900	0.00079700
C	3.58309600	-1.19498100	0.07478700
C	3.57643800	1.20097100	-0.08590800
C	2.18753500	1.18373700	-0.08072200
C	2.19407700	-1.18504000	0.07822500
F	5.60810900	0.00821600	-0.01175400
F	4.24081900	2.35514200	-0.16475100
F	1.55397800	2.35943200	-0.15758500
F	1.56721300	-2.36401600	0.15842700
F	4.25392100	-2.34569300	0.14956200
I	-0.65533700	-0.00637300	0.00588200
N	-3.50485600	0.00032000	0.00867300
C	-3.96307300	0.51417000	1.31073900
H	-5.05703300	0.67968500	1.28069700
H	-3.49387300	1.49014400	1.46176700
C	-3.97353300	-1.38056800	-0.19915700
H	-5.06846600	-1.42810200	-0.04444100
H	-3.51192500	-2.00304800	0.57210600
C	-3.96129400	0.87455400	-1.08545700
H	-5.05650600	0.77319000	-1.20926200
H	-3.49834100	0.51343600	-2.00789600
C	-3.63363000	-1.97907700	-1.56004200
H	-3.85241800	-3.05213200	-1.53752600
H	-2.57044500	-1.86123800	-1.79701700
H	-4.22134800	-1.54388400	-2.37397700
C	-3.61051300	2.34978100	-0.92204900
H	-4.19859500	2.84182500	-0.14146900
H	-3.82078400	2.86818600	-1.86382800

H	-2.54760900	2.48864600	-0.69553600
C	-3.62320600	-0.36805100	2.50750600
H	-2.56217100	-0.64113600	2.51753000
H	-4.21760500	-1.28610500	2.54035900
H	-3.83275200	0.18913400	3.42702400

Zero-point correction= 0.260650 (Hartree/Particle)
Thermal correction to Energy= 0.281813
Thermal correction to Enthalpy= 0.282757
Thermal correction to Gibbs Free Energy= 0.207706
Sum of electronic and zero-point Energies= -1317.469339
Sum of electronic and thermal Energies= -1317.448176
Sum of electronic and thermal Enthalpies= -1317.447232
Sum of electronic and thermal Free Energies= -1317.522283
E(RwB97XD/6-311+G(2d,p)+def2-TZVPD(I)) = -1318.02446700

wb97xd-F5PhI-C-HEXANE

C	-0.51311200	-0.70827800	0.75239300
C	-0.25725400	-0.59910200	3.53815100
C	0.36668000	-1.56245000	1.40390900
C	-1.26517400	0.20056400	1.48497100
C	-1.13306300	0.24859500	2.86678100
C	0.48777600	-1.50252500	2.78630100
F	-0.63588400	-0.76078100	-0.56963900
F	-2.10918900	1.01964700	0.86070900
F	-1.87115500	1.13338200	3.53625000
F	1.34227400	-2.33717700	3.37707200
F	1.08833000	-2.43425800	0.70238900
I	-0.06584900	-0.51743400	5.61588400

Zero-point correction= 0.050258 (Hartree/Particle)
Thermal correction to Energy= 0.060490
Thermal correction to Enthalpy= 0.061434
Thermal correction to Gibbs Free Energy= 0.012634
Sum of electronic and zero-point Energies= -1025.324378
Sum of electronic and thermal Energies= -1025.314146
Sum of electronic and thermal Enthalpies= -1025.313202
Sum of electronic and thermal Free Energies= -1025.362002
E(RwB97XD/6-311+G(2d,p)+def2-TZVPD(I)) = -1025.59167395

wb97xd-NEt3-C-HEXANE

N	0.00110500	0.00009400	0.06463200
C	0.51009600	1.30154500	0.49700900
H	0.83143900	1.26415500	1.55658400
H	-0.31268000	2.02157600	0.45132600
C	0.87355200	-1.09220100	0.49509200
H	0.68509500	-1.34920500	1.55618100
H	1.90902400	-0.74210400	0.44350400
C	-1.38087800	-0.20955500	0.49554000
H	-1.51003900	0.08642100	1.55530200
H	-1.59380400	-1.28179700	0.44846700
C	0.74822700	-2.33589100	-0.37791600
H	1.44249300	-3.11153600	-0.03380000
H	0.98597800	-2.09185800	-1.41878400

H	-0.26027700	-2.76288000	-0.35325400
C	-2.39546000	0.51588700	-0.38125000
H	-2.25993000	1.60273200	-0.36333800
H	-3.41439100	0.30538200	-0.03552200
H	-2.30355200	0.18162900	-1.42012700
C	1.64487000	1.82018400	-0.37926000
H	1.30924200	1.90842100	-1.41804500
H	2.51957400	1.16096600	-0.36221500
H	1.97031500	2.80803300	-0.03239500

Zero-point correction= 0.208248 (Hartree/Particle)
Thermal correction to Energy= 0.217581
Thermal correction to Enthalpy= 0.218525
Thermal correction to Gibbs Free Energy= 0.174412
Sum of electronic and zero-point Energies= -292.133985
Sum of electronic and thermal Energies= -292.124652
Sum of electronic and thermal Enthalpies= -292.123707
Sum of electronic and thermal Free Energies= -292.167821
E(RwB97XD/6-311+G(2d,p)+def2-TZVPD(I)) = -292.418954105

References

[1] Frisch, M. J.; Trucks, G. W.; Schlegel, H. B.; Scuseria, G. E.; Robb, M. A.; Cheeseman, J. R.; Scalmani, G.; Barone, V.; Petersson, G. A.; Nakatsuji, H.; Li, X.; Caricato, M.; Marenich, A. V.; Bloino, J.; Janesko, B. G.; Gomperts, R.; Mennucci, B.; Hratchian, H. P.; Ortiz, J. V.; Izmaylov, A. F.; Sonnenberg, J. L.; Williams-Young, D.; Ding, F.; Lipparini, F.; Egidi, F.; Goings, J.; Peng, B.; Petrone, A.; Henderson, T.; Ranasinghe, D.; Zakrzewski, V. G.; Gao, J.; Rega, N.; Zheng, G.; Liang, W.; Hada, M.; Ehara, M.; Toyota, K.; Fukuda, R.; Hasegawa, J.; Ishida, M.; Nakajima, T.; Honda, Y.; Kitao, O.; Nakai, H.; Vreven, T.; Throssell, K.; Montgomery, J. A., Jr.; Peralta, J. E.; Ogliaro, F.; Bearpark, M. J.; Heyd, J. J.; Brothers, E. N.; Kudin, K. N.; Staroverov, V. N.; Keith, T. A.; Kobayashi, R.; Normand, J.; Raghavachari, K.; Rendell, A. P.; Burant, J. C.; Iyengar, S. S.; Tomasi, J.; Cossi, M.; Millam, J. M.; Klene, M.; Adamo, C.; Cammi, R.; Ochterski, J. W.; Martin, R. L.; Morokuma, K.; Farkas, O.; Foresman, J. B.; Fox, D. J. *Gaussian 16 Rev. C.01*, Wallingford, CT, 2016.
